# Functional Characterization of the P‐Type ATPases ATP13A3 and ATP13A4 as Plasma Membrane Polyamine Transporters

**DOI:** 10.1096/fj.202603205RR

**Published:** 2026-10-13

**Authors:** Haruka Morimoto, Tomoya Yasujima, Mione Yamasaki, Takahiro Yamashiro, Hiroaki Yuasa

**Affiliations:** ^1^ Department of Biopharmaceutics, Graduate School of Pharmaceutical Sciences Nagoya City University Nagoya Japan

**Keywords:** ATP13A3, ATP13A4, plasma membrane transport, polyamine, P‐type ATPase

## Abstract

Polyamines are ubiquitous polycations involved in diverse cellular functions, including proliferation, differentiation, and survival. While endogenous biosynthesis can maintain the intracellular levels of polyamines, uptake becomes critical during high metabolic demand. Despite the evidence for ATP‐dependent polyamine uptake systems in the plasma membrane, their molecular characteristics remain unclear. We found that two human P5B ATPases—ATP13A3 and ATP13A4—were localized to the plasma membrane and directly mediated the uptake of putrescine and spermidine in an ATP‐dependent manner. Using ATP13A3 and ATP13A4 heterologously expressed in COS‐7 cells, we demonstrated that they mediate the saturable, ATP‐dependent uptake of polyamines with distinct kinetic profiles, consistent with the characteristics of known uptake systems in mammalian cells. Site‐directed mutagenesis of the conserved ATPase motif abolished uptake, confirming the requirement of ATP hydrolysis. The immunofluorescence analysis in COS‐7 cells transiently expressing FLAG‐tagged ATP13A3 or ATP13A4 revealed that both transporters localize to the plasma membrane. RNA interference in HepG2 cells to suppress endogenous ATP13A3 and ATP13A4 reduced polyamine uptake, supporting the physiological relevance of these transporters. It was also found that ATP13A3 is ubiquitously expressed and has moderate affinity for polyamines, whereas ATP13A4 is more specific tissue‐restricted and has higher affinity for polyamines, particularly spermidine. These findings establish ATP13A3 and ATP13A4 as the first P‐type ATPases shown to transport small organic solutes for cellular uptake at the plasma membrane. This study elucidates the roles of these P‐type ATPases in polyamine homeostasis. Furthermore, given the emerging associations of ATP13A3 and ATP13A4 with neurological and metabolic disorders, these transporters are promising therapeutic targets in polyamine‐related diseases.

## Graphical abstract

ATP13A3 and ATP13A4 mediate the plasma membrane uptake of the polyamines, putrescine and spermidine, in an ATP‐dependent manner. ATP13A3 is broadly expressed across human tissues, whereas ATP13A4 is expressed at relatively high levels in the brain and lung. These findings establish ATP13A3 and ATP13A4 as key components of the mammalian plasma membrane polyamine transport system.

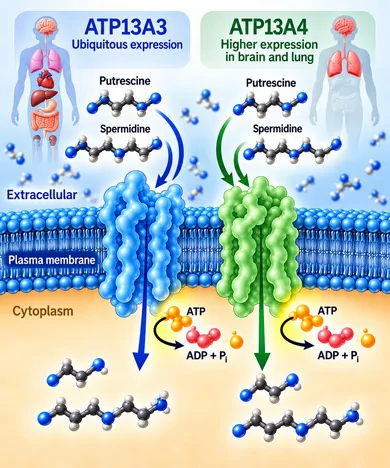

## 1 Introduction

Polyamines such as putrescine, spermidine, and spermine are small polycationic naturally occurring compounds that play indispensable roles in cellular homeostasis and a wide array of physiological processes [1, 2]. Because of their cationic nature, polyamines bind electrostatically to negatively charged biomolecules, including nucleic acids, acidic proteins, and phospholipids, modulating essential cellular activities such as chromatin remodeling, mRNA translation, and membrane stability [3, 4, 5]. Polyamines also exert pleiotropic effects on cell proliferation, differentiation, apoptosis, immune responses, and organismal aging [6, 7, 8]. Aberrations in polyamine metabolism have been implicated in pathological conditions, including neoplasia, neurodegenerative disorders, and immunological dysregulation.

Several pathways regulate the biosynthesis, catabolism, efflux, and uptake of polyamines to maintain their optimal concentrations inside cells [9]. While intracellular biosynthesis provides basic levels of polyamines, uptake becomes crucial under conditions of high metabolic demand and stress [10, 11]. Radiolabeled polyamine uptake studies have identified ATP‐dependent polyamine transport systems at the plasma membrane [12, 13]. These systems have characteristics that are inconsistent with simple diffusion or endocytosis, exhibiting saturable kinetics and susceptibility to inhibitors. However, the molecular characteristics of plasma membrane polyamine transporters are unclear.

Polyspecific organic cation transporters have been suggested to be potential polyamine transporters [14, 15, 16]. However, these transporters generally lack strict ATP dependence and display broad substrate promiscuity with modest specificity to polyamines. Thus, these transporters are unlikely to account for the ATP‐dependent polyamine uptake observed in mammalian cells. This realization shifted attention toward alternative transporter families that might fulfill the stringent functional criteria derived from previous uptake studies.

P5B ATPases, which belong to P‐type ATPases, are potential mediators of polyamine transport. P‐type ATPases are ATP‐driven pumps that translocate inorganic cations (e.g., Na^+^, K^+^, Ca^2+^, H^+^, and Cu^2+^) or phospholipids across biological membranes. However, the P5B subclass appears to have diverged to handle unconventional substrates. Notably, ATP13A2, a P5B ATPase, has been extensively studied due to its link to neurodegenerative diseases such as Kufor‐Rakeb syndrome and early‐onset Parkinson's disease [17, 18, 19]. ATP13A2 localizes to lysosomes and late endosomes, where it exports polyamines from the organelle lumen to the cytosol [20, 21], alleviating polyamine‐induced lysosomal stress and preserving organellar integrity.

ATP13A3 and ATP13A4 are less studied than other P5B ATPases but have garnered attention due to their potential association with diseases. ATP13A3 is ubiquitously expressed and is genetically linked to pulmonary arterial hypertension—a vascular disease characterized by endothelial hyperproliferation and remodeling [22]. ATP13A4 is more specific tissue‐restricted and is associated with neurodevelopmental disorders, including autism spectrum disorder and intellectual disability [23, 24]. Although emerging studies have suggested the intracellular localization of ATP13A3 and ATP13A4 and posited their involvement in organellar polyamine export akin to ATP13A2, such assertions have predominantly relied on indirect methodologies, including fluorescent imaging with polyamine analogs conjugated to fluorophores [25, 26]. Thus, the physiological relevance of ATP13A3 and ATP13A4 in polyamine transport is unclear.

This study evaluated the subcellular localization and functions of human ATP13A3 and ATP13A4. We performed uptake assays for transport characterization, including ATP dependence assessments and kinetic profiling, in heterologous expression systems using COS‐7 cells to determine whether these transporters mediate the uptake of polyamines across the plasma membrane in a directly ATP‐dependent manner, using putrescine and spermidine as representative substrates. We also examined their tissue distribution patterns to infer physiological specialization. This study should help elucidate the molecular mechanisms of polyamine uptake in mammalian systems.

## 2 Materials and Methods

### 2.1 Materials and Cell Lines

A comprehensive list of materials and cell lines used in this study is provided in Table S1.

### 2.2 Cell Culture

COS‐7, HepG2, and A549 cells were cultured in DMEM supplemented with 10% fetal bovine serum and 1% penicillin/streptomycin at 37°C in a humidified atmosphere containing 5% CO_2_, as described previously [27].

### 2.3 Plasmids

The cDNA encoding human ATP13A3 (GenBank Accession No. NM_024524.4) was cloned using a reverse transcription‐PCR method described previously [28]. For this purpose, total RNA isolated from human liver (BioChain Institute, Newark, CA, USA) was reverse transcribed into cDNA using oligo (dT) primers and ReverTra Ace reverse transcriptase (Toyobo, Osaka, Japan). ATP13A3 cDNA was amplified by PCR using KOD ONE DNA Polymerase (Toyobo), followed by a second PCR to incorporate restriction sites. The cDNAs of human ATP13A1, ATP13A2, and ATP13A5 were cloned from total RNA isolated from human brain (BioChain Institute), and the cDNA of human ATP13A4 was cloned from total RNA isolated from human lung (BioChain Institute). The GenBank Accession Nos. are NM_020410.3, NM_022089.4, NM_032279.4, and NM_198505.4, respectively, for ATP13A1, ATP13A2, ATP13A4, and ATP13A5. Primer sequences are listed in Table S2.

The cDNA products were subcloned into the pCI‐neo vector (Promega, Madison, WI, USA) for use in the determination of calibration curves for the absolute quantification of mRNA levels and the preparation of constructs FLAG‐tagged at the C‐terminus. All constructs were sequence‐verified using an automated sequencer (ABI PRISM 3130, Applied Biosystems, Foster City, CA, USA), as described previously [29].

Site‐directed mutagenesis of ATP13A3 and ATP13A4 was performed using the PrimeSTAR Mutagenesis Basal Kit (Takara Bio, Kusatsu, Japan) and KOD ONE Polymerase. The primers used for mutagenesis are listed in Table S3.

The cDNAs for ATP13A3 and ATP13A4 with FLAG‐tag inserted into the extracellular loop between the ninth and tenth transmembrane domains were prepared using the PrimeSTAR Mutagenesis Basal Kit and KOD ONE Polymerase. The primers used for FLAG‐tag insertion are listed in Table S4.

### 2.4 Preparation of Transiently Transfected COS‐7 Cells

COS‐7 cells (3.0 × 10^5^ cells/mL, 0.5 mL/well) were seeded in 24‐well plates and transfected with a designated plasmid (1 μg/well), using polyethylenimine Max (2 μg/well, Polysciences, Warrington, PA, USA). Cells were cultured for 48 h after transfection. Mock cells were prepared by transfecting the empty pCI‐neo vector.

### 2.5 Western Blot Analysis

Protein expression in transiently transfected COS‐7 cells cultured in 24‐well plates was measured by Western blotting. Cells were lysed in buffer (50 mM Tris–HCl, pH 8.0, 1% SDS, 4 M urea, 1 mM EDTA, 150 mM NaCl), and lysates were processed as described previously [28]. Primary antibodies were anti‐FLAG mouse monoclonal antibody (1:1000; FUJIFILM Wako, Osaka, Japan) and anti‐β‐actin mouse monoclonal antibody (1:1000; Proteintech, Rosemont, IL, USA), and horseradish peroxidase‐conjugated goat anti‐mouse IgG (1:10000; Jackson ImmunoResearch, West Grove, PA, USA) was used as a secondary antibody.

### 2.6 Uptake Assays

Uptake assays were performed as previously described [30]. Cells were seeded in 24‐well plates, and uptake was measured in Hanks' balanced salt solution supplemented with 10 mM MES (for pH ≤ 6.5) or 10 mM HEPES (for pH ≥ 7.0), and added with [^3^H]putrescine (80 Ci/mmol, American Radiolabeled Chemicals, St. Louis, MO, USA) or [^3^H]spermidine (59.6 Ci/mmol, PerkinElmer, Waltham, MA, USA) as a radiolabeled substrate. Preincubation was conducted using the substrate‐free solution. In inhibition studies, [^3^H]putrescine or [^3^H]spermidine was used at a tracer concentration of 1 nM, which was substantially lower than the *K*_m_ values determined for ATP13A3 and ATP13A4, to enable sensitive detection of the inhibition of their mediated uptake under the condition where they operate most efficiently with minimal occupancy by the substrate. Test compounds were added at a high concentration of 100 μM, or 50 μM for decynium‐22, to screen for compounds capable of interacting with the transporters. The radiolabeled substrate and each test compound were added simultaneously at the initiation of uptake. Uptake was normalized to total cellular protein quantified using the Protein Assay BCA Kit (FUJIFILM Wako) with BSA as the standard. Specific uptake was determined by subtracting the uptake in mock‐transfected cells from that in transfected cells.

### 2.7 Immunofluorescence Staining

Transiently transfected COS‐7 cells were seeded in 24‐well plates (3.0 × 10^5^ cells/mL, 0.5 mL/well) and cultured for 48 h. Cells were washed with ice‐cold PBS and fixed in methanol (−20°C for 10 min) for immunofluorescence staining. Alternatively, cells were stained without fixation. Cells were blocked with 1 mg/mL BSA in Opti‐MEM (room temperature for 1 h) and then incubated with anti‐FLAG mouse monoclonal antibody (1:500 dilution) in Opti‐MEM for 2 h. Following PBS wash, cells were incubated with Alexa Fluor Plus 488–conjugated goat anti‐mouse IgG (1:500; Thermo Fisher Scientific, Waltham, MA, USA) for 1 h. Images were acquired using a fluorescence microscope (BZ‐X800, Keyence, Osaka, Japan).

### 2.8 Quantitative Real‐Time PCR Analysis of mRNA

Total RNA extracted from various human tissues and cell lines was reverse transcribed to obtain cDNA, using ReverTra Ace reverse transcriptase, and quantitative real‐time PCR was performed using the Taq Pro Universal SYBR qPCR Master Mix (Vazyme Biotech, Nanjing, China) on a CFX Connect Real‐Time PCR System (Bio‐Rad Laboratories, Hercules, CA, USA) with gene‐specific primers (Table S5). Donor information for the human tissue total RNA samples (BioChain Institute) is provided in Table S1. Absolute quantification was performed by generating standard curves using pCI‐neo‐based plasmids carrying ATP13A3 or ATP13A4 cDNA. Expression levels were normalized to those of ubiquitin C (tissue samples) or GAPDH (cell lines).

### 2.9 Knockdown Assays

HepG2 and A549 cells (1.0 × 10^5^ cells/mL, 0.5 mL/well) were seeded in 24‐well plates and cultured for 12 h. Cells were transfected with Silencer Select siRNAs (5 pmol/well, Thermo Fisher Scientific) targeting ATP13A3 or ATP13A4, using Lipofectamine RNAiMAX (1.5 μL/well), and cultured for 4 days post‐transfection. The siRNA sequences are listed in Table S6. Cells transfected with negative control siRNA served as the controls. Putrescine uptake and mRNA expression were assessed as described above.

### 2.10 Data Analysis

The saturable transport of putrescine and spermidine was analyzed using the Michaelis–Menten equation: *v* = *V*_max_ × *s*/(*K*_m_ × *s*), where *V*_max_ is the maximum transport rate and *K*_m_ is the Michaelis constant. *V*_max_ and *K*_m_ were estimated by fitting this equation to the experimental profile of the uptake rate (*v*) versus the substrate concentration (*s*) using a non‐linear least‐squares regression analysis program (WinNonlin; Pharsight Corp., Mountain View, CA).

When *s* is much smaller than *K*_m_ (*s* < *K*_m_), *v* in the presence of a competitive inhibitor can be described using the following equation: *v* = *v*_0_/(1 + (*i*/IC_50_)^*n*^), where IC_50_ is the half‐maximal inhibitory concentration, *i* is the inhibitor concentration, and *n* is the Hill coefficient. IC_50_ was estimated together with *n* and *v* in the absence of inhibitors (*v*_0_) by fitting this equation to the experimental profile of *v* versus *i*.

Statistical comparisons between two groups were performed using Student's *t*‐test. Comparisons among three or more groups were performed using one‐way analysis of variance (ANOVA) followed by Dunnett's multiple‐comparison test or Bonferroni's multiple‐comparison test, as described in the figure legends. Data are presented as means ± standard deviations. *p* values < 0.05 were considered statistically significant.

## 3 Results

### 3.1 Polyamine Transport by ATP13As in Transiently Transfected COS‐7 Cells

To determine polyamine transport by ATP13A isoforms, COS‐7 cells were transiently transfected with plasmids encoding human ATP13A1, ATP13A2, ATP13A3, ATP13A4, or ATP13A5, each fused with a FLAG epitope tag at the C‐terminus and uptake assays were performed at pH 7.4 using [^3^H]putrescine (1 nM) and [^3^H]spermidine (1 nM), as shown in Figure 1. In ATP13A3‐transfected and ATP13A4‐transfected cells, the uptakes of putrescine and spermidine were significantly greater than those in mock‐transfected control cells, indicating that ATP13A3 and ATP13A4 mediate their transport. In contrast, the other transporters exerted no detectable effect on their uptake. Previous studies have reported that lysosomal membrane‐localized ATP13A2 is involved in an elevation of the cellular uptake of these polyamines. Given its intracellular localization, the absence of the uptake activity in ATP13A2‐transfected cells may reflect the limited accessibility of these polyamines to the lysosomal compartment, particularly under the short incubation period used in this study. In contrast, the robust uptake activity of ATP13A3 and ATP13A4 suggests that these isoforms localize to the plasma membrane and facilitate the cellular uptake transport of these polyamines. Western blots showed that all five FLAG‐tagged isoforms were highly and comparably expressed, indicating that differences in the change in uptake were unaffected by the expression levels. These findings indicate that, in the ATP13A subfamily, ATP13A3 and ATP13A4 mediate polyamine uptake at the plasma membrane in transfected COS‐7 cells, functionally distinguishing them from the other family members.

**FIGURE 1:**
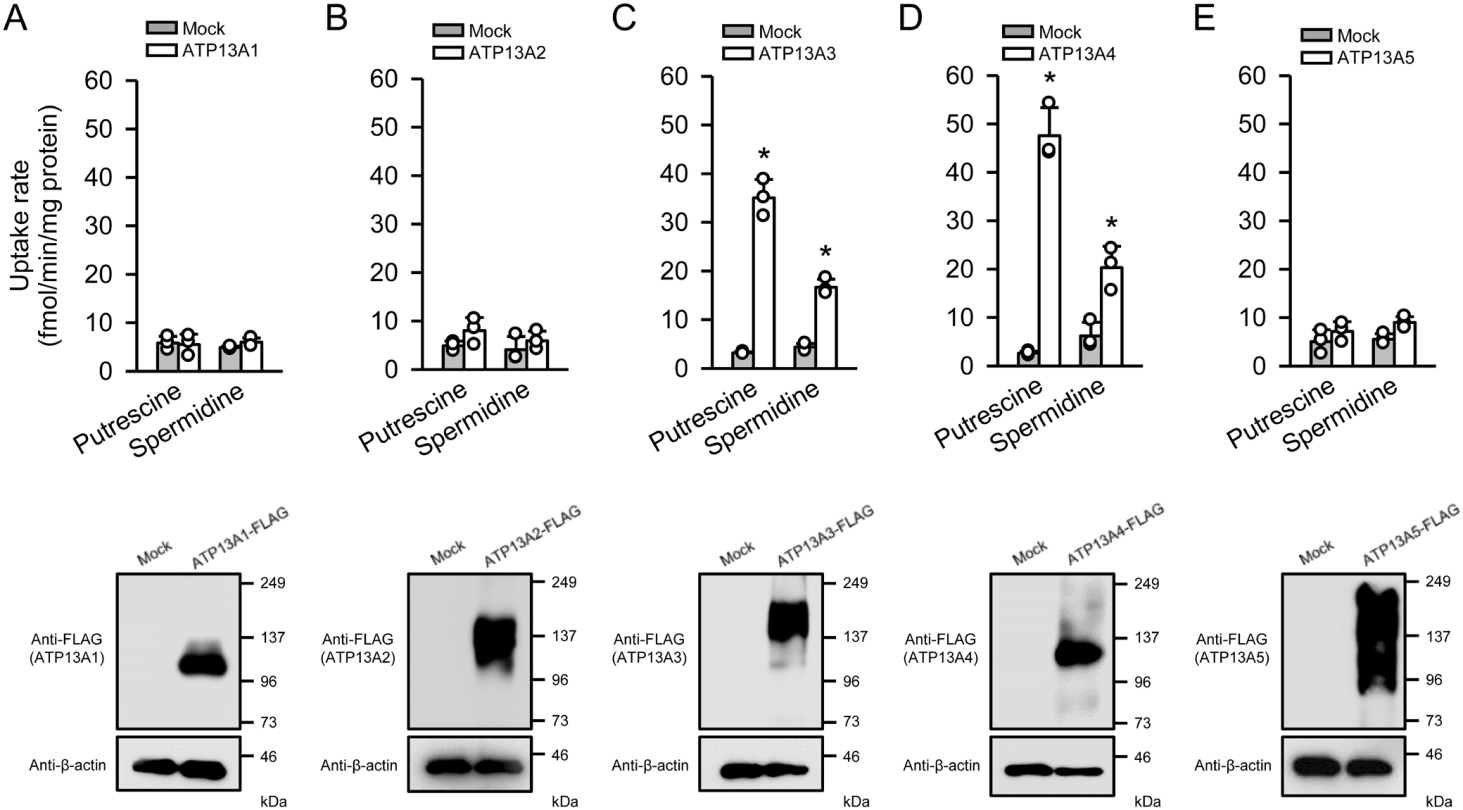
Uptake of putrescine and spermidine in COS‐7 cells transiently transfected with ATP13A family transporters. The uptake of [^3^H]putrescine (1 nM) and [^3^H]spermidine (1 nM) was measured for 5 min at pH 7.4 and 37°C in cells transfected with FLAG‐tagged ATP13A1 (A), ATP13A2 (B), ATP13A3 (C), ATP13A4 (D), or ATP13A5 (E), and in mock‐transfected cells. Protein expression was assessed by Western blotting using an anti‐FLAG antibody, with β‐Actin serving as a loading control. Data are presented as means ± standard deviations from three biological replicates (*N* = 3), each of which was performed in duplicate (*n* = 2) as technical replication. Statistical significance was determined using Student's *t*‐test. *p < 0.05 vs. the mock control.

### 3.2 Localization of ATP13A3 and ATP13A4

The subcellular localization of ATP13A3 and ATP13A4 was evaluated by immunofluorescence staining under permeabilized and non‐permeabilized conditions using two engineered constructs of each transporter (Figure 2A), similar to previous studies assessing membrane protein topology [31, 32]. One set of constructs featured a FLAG epitope fused to the intracellular C‐terminus (FLAG_C‐terminus_), while the second set, referred to as FLAG_outside_, had a FLAG epitope inserted into an extracellular loop between the ninth and tenth transmembrane domains. The position of this loop was predicted based on transmembrane topology analysis using DeepTMHMM, a deep‐learning tool for membrane protein topology prediction. This dual‐construct strategy was designed to distinguish between intracellular and plasma membrane localization, such that the FLAG_C‐terminus_ epitope would only be accessible to antibodies after permeabilization in both. In contrast, the FLAG_outside_ epitope would be detectable without permeabilization only if the transporter were localized to the plasma membrane.

**FIGURE 2:**
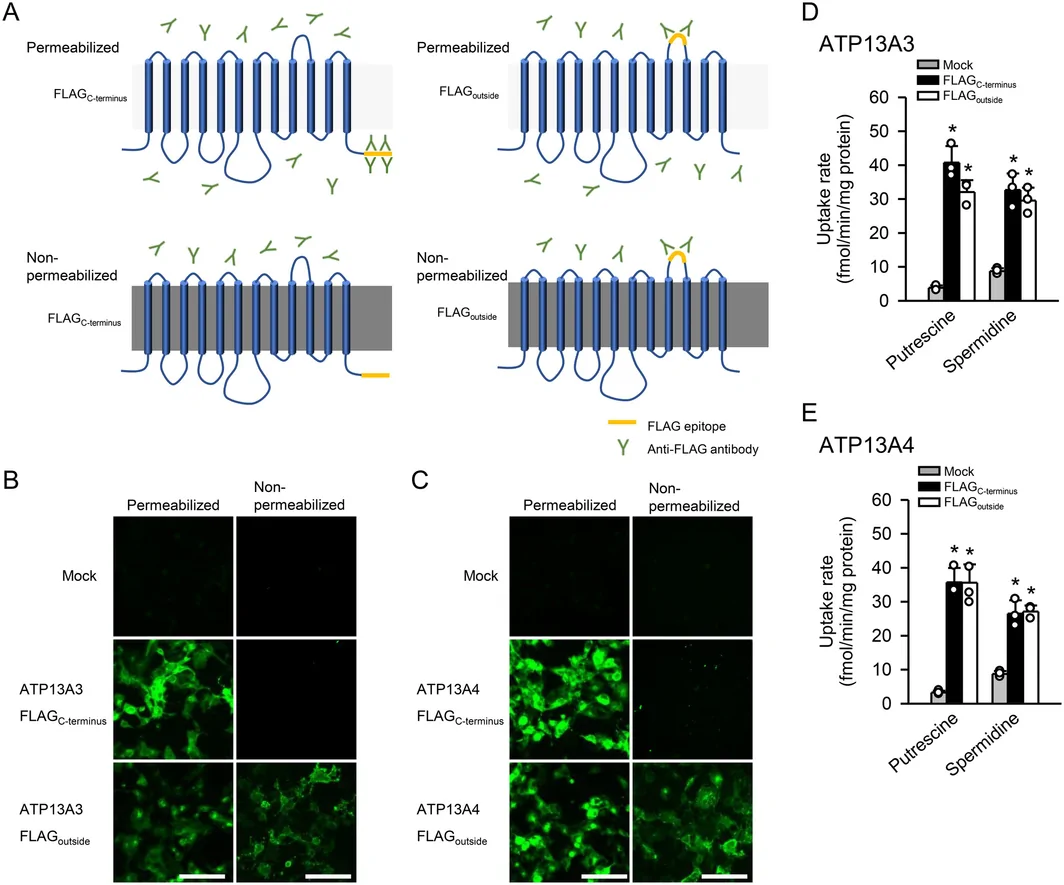
Expression of ATP13A3 and ATP13A4 at the plasma membrane in transiently transfected COS‐7 cells. (A) Schematic representation of ATP13A3 and ATP13A4 constructs tagged with a FLAG epitope at the C‐terminus and within the extracellular loop between the ninth and tenth transmembrane domains. Immunofluorescence analysis of cells transfected with FLAG‐tagged ATP13A3 (B) and ATP13A4 (C). The cells were stained under two conditions to evaluate the subcellular localization of the FLAG tag: (1) with digitonin‐induced permeabilization, which allows for the targeting of intracellular and extracellular epitopes by antibodies; (2) without permeabilization, where only extracellular epitopes are accessible. Scale bar: 100 μm. The uptake of [^3^H]putrescine (1 nM) and [^3^H]spermidine (1 nM) was measured for 5 min at pH 7.4 and 37°C in cells transfected with FLAG‐tagged ATP13A3 (D) or ATP13A4 (E), and in mock‐transfected cells. Data are presented as means ± standard deviations from three biological replicates (*N* = 3), each of which was performed in duplicate (*n* = 2) as technical replication. Statistical significance was determined using analysis of variance followed by Dunnett's test. *p < 0.05 vs. the mock control.

Cells expressing the FLAG_C‐terminus_ of ATP13A3 exhibited fluorescence only after permeabilization, consistent with the intracellular presence of the epitope. In contrast, cells expressing the FLAG_outside_ showed strong surface fluorescence even without permeabilization, indicating that ATP13A3 was localized to the plasma membrane (Figure 2B). Cells expressing the FLAG_C‐terminus_ of ATP13A4 exhibited fluorescence only after permeabilization, whereas cells expressing the FLAG_outside_ showed strong fluorescence without permeabilization, confirming that ATP13A4 was also localized to the plasma membrane (Figure 2C). For both ATP13A3 (Figure 2D) and ATP13A4 (Figure 2E), the uptake activity of the FLAG_outside_ for [^3^H]putrescine (1 nM) and [^3^H]spermidine (1 nM) was confirmed to be comparable to that of the FLAG_C‐terminus_, as indicated by the similarly elevated uptake of each polyamine in cells expressing the FLAG_outside_ and FLAG_C‐terminus_, compared with the uptake in mock cells. These results demonstrate that ATP13A3 and ATP13A4 are localized to the plasma membrane in transfected COS‐7 cells, supporting their role in mediating the uptake of extracellular polyamines.

### 3.3 Characterization of ATP13A3‐Mediated Putrescine Transport in Transiently Transfected COS‐7 Cells

Putrescine transport by ATP13A3 was evaluated by performing uptake assays in COS‐7 cells transiently transfected with ATP13A3 for detailed functional characterization. The uptake of [^3^H]putrescine (1 nM) was significantly elevated in ATP13A3‐transfected cells relative to mock control cells at pH 7.4, indicating substantial putrescine transporter activity (Figure 3A). The elevated portion of the uptake, which was ATP13A3‐specific, decreased markedly under the ATP‐depleted condition, suggesting ATP‐dependent putrescine transport. We also examined the effect of the modulation of the ATPase domain responsible for ATP hydrolysis by mutating D498, which is the conserved phosphorylatable aspartate residue in the P domain of P‐type ATPases and is essential for the ATPase catalytic cycle [33, 34]. As shown in Figure 3B, the D498N mutant, which has previously been reported as a catalytically inactive/transport‐deficient mutant [25], exhibited markedly reduced ATP13A3‐specific uptake (Figure 3B), supporting the requirement of an intact ATPase catalytic cycle for putrescine transport.

**FIGURE 3:**
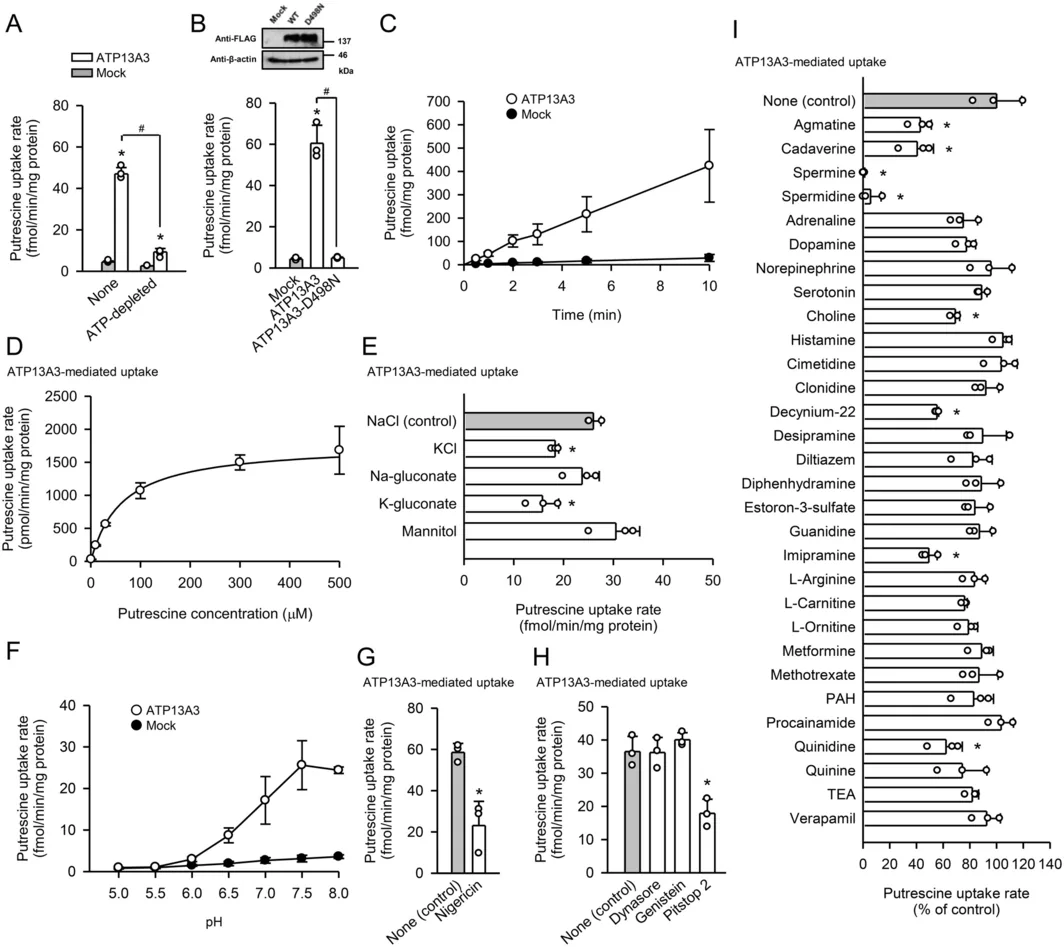
Functional characteristics of ATP13A3‐mediated putrescine transport in transiently transfected COS‐7 cells. (A) Effect of ATP depletion. The uptake of [^3^H]putrescine (1 nM) was measured for 5 min at pH 7.4 and 37°C in ATP13A3‐transfected cells and in mock‐transfected cells with or without ATP depletion. ATP depletion was induced by preincubation (20 min) in the presence of sodium azide (10 mM), sodium fluoride (10 mM), and 3‐O‐methyl‐D‐glucopyranose (25 mM). (B) Effect of D498N mutation in the ATPase domain. Expression of the wild type (WT) and D498N mutant of FLAG‐tagged ATP13A3 in cells transfected with them was confirmed by Western blotting using an anti‐FLAG antibody, with β‐Actin serving as a loading control (upper panel). The uptake of [^3^H]putrescine (1 nM) was measured for 5 min at pH 7.4 and 37°C in cells transfected with the WT or D498N mutant of FLAG‐tagged ATP13A3 and in mock‐transfected cells (lower panel). (C) Time course of putrescine uptake. The uptake of [^3^H]putrescine (1 nM) was measured at pH 7.4 and 37°C in ATP13A3‐transfected cells and in mock‐transfected cells. (D) Concentration dependence. The specific uptake of [^3^H]putrescine by ATP13A3 in its transfected cells was evaluated for 5 min at pH 7.4 and 37°C. The *V*_max_ and *K*_m_ were 1780 ± 220 pmol/min/mg protein and 62.8 ± 5.5 μM, respectively. (E) Effect of ionic composition. The specific uptake of [^3^H]putrescine (1 nM) by ATP13A3 in its transfected cells was evaluated for 5 min at pH 7.4 and 37°C, where NaCl in the control medium was replaced as indicated. (F) Effect of extracellular pH. The uptake of [^3^H]putrescine (1 nM) was measured for 5 min at 37°C in ATP13A3‐transfected cells and in mock‐transfected cells. (G) Effect of intracellular acidification. The specific uptake of [^3^H]putrescine (1 nM) by ATP13A3 in its transfected cells was evaluated for 5 min at pH 7.4 and 37°C after pretreatment with nigericin (10 μM) for 5 min to induce intracellular acidification. (H) Effect of endocytosis inhibitors. The specific uptake of [^3^H]putrescine (1 nM) by ATP13A3 in its transfected cells was evaluated for 5 min at pH 7.4 and 37°C after pretreatment with inhibitors (50 μM for pitstop 2 and 100 μM for the others) for 30 min. (I) Effect of various compounds. The specific uptake of [^3^H]putrescine (1 nM) by ATP13A3 in its transfected cells was evaluated for 5 min at pH 7.4 and 37°C in the presence or absence of test compounds (50 μM for decynium‐22 and 100 μM for the others). PAH, *p*‐aminohippuric acid; TEA, tetraethylammonium. Data are presented as means ± standard deviations from three biological replicates (*N* = 3), each of which was performed in duplicate (*n* = 2) as technical replication. Statistical significance was determined using analysis of variance followed by Bonferroni's test (A, B) or Dunnett's test (E, H, I), or using Student's *t*‐test (G). *p < 0.05 vs. the control or mock group. #*p* < 0.05 vs. the indicated group.

Time‐course analysis revealed increasing putrescine uptake in proportion to time up to 10 min in ATP13A3‐transfected cells, with minimal uptake in mock cells (Figure 3C). Based on these findings, a 5‐min incubation period was selected for uptake evaluations in subsequent functional analyses. Kinetic analysis demonstrated saturable ATP13A3‐mediated putrescine transport, conforming to the Michaelis–Menten model with a *V*_max_ of 1780 pmol/min/mg protein and a *K*_m_ of 62.8 μM (Figure 3D). To investigate ion dependency, NaCl was replaced with equimolar KCl, Na‐gluconate, K‐gluconate, or mannitol in the uptake buffer. Although KCl and K‐gluconate reduced ATP13A3‐specific putrescine uptake slightly, Na‐gluconate and mannitol had no effect (Figure 3E). These results suggest that Na^+^, K^+^, or Cl^−^ practically has no major influence on the putrescine transport function, although K^+^ may impair the function slightly. Putrescine uptake by ATP13A3 was, however, found to be pH‐sensitive, as putrescine uptake increased with an increase in pH, reaching a maximal level at around pH 7.5 in ATP13A3‐transfected cells, whereas it was low and independent of pH in mock cells (Figure 3F). Pretreatment with nigericin in the mannitol‐based buffer to induce intracellular acidification, which could enhance uptake if H^+^/substrate exchange were the mechanism of transport, significantly reduced ATP13A3‐specific putrescine uptake (Figure 3G), indicating that H^+^/putrescine exchange is not a mechanism for the pH‐sensitive uptake. To examine the potential involvement of endocytic processes, cells were treated with clathrin‐mediated endocytosis inhibitors (dynasore and pitstop 2) and a caveolae‐mediated endocytosis inhibitor (genistein). As shown in Figure 3H, dynasore did not affect the specific uptake of putrescine by ATP13A3. Although pitstop 2 significantly reduced the specific uptake, the absence of a detectable effect of dynasore indicates that the inhibition by pitstop 2 is likely due to off‐target effects. These results suggest that endocytosis is not likely to be involved in ATP13A3‐specific putrescine uptake.

The profile of the effect of various compounds (50 μM for decynium‐22 and 100 μM for the others) on the specific uptake of putrescine (1 nM) by ATP13A3 is shown in Figure 3I. Particularly strong inhibition was observed with polyamines (spermidine and spermine). Polyamine analogs (agmatine and cadaverine) also exerted inhibitory effects, but to lesser extents. Choline exerted a weak but statistically significant inhibitory effect. Among cationic drugs, decynium‐22, imipramine, and quinidine moderately inhibited the specific uptake. The IC_50_ values of the compounds that caused strong inhibition (spermidine, spermine, agmatine, and cadaverine) are shown in Table 1.

**TABLE 1:** IC_50_ values of polyamines and polyamine analogs as inhibitors for polyamine transport by ATP13A3 and ATP13A4.

Inhibitor	IC_50_ (μM)			
	ATP13A3		ATP13A4	
	Putrescine	Spermidine	Putrescine	Spermidine
Putrescine	—	262 ± 58	—	11.2 ± 6.5
Spermidine	21.3 ± 2.5	—	6.08 ± 7.27	—
Spermine	6.08 ± 4.10	5.59 ± 4.02	5.82 ± 3.81	15.7 ± 9.1
Agmatine	304 ± 180	8540 ± 1600	10.7 ± 0.6	15.7 ± 10.5
Cadaverine	273 ± 48	1430 ± 370	42.5 ± 2.0	11.7 ± 6.3

*Note:* The values are means with standard deviations (*n* = 3).

### 3.4 Characterization of ATP13A3‐Mediated Spermidine Transport in Transiently Transfected COS‐7 Cells

The functional characteristics of ATP13A3 were also assessed for spermidine transport by uptake assays similar to those for putrescine transport. At pH 7.4, ATP13A3‐transfected cells exhibited significantly higher uptake of [^3^H]spermidine (1 nM) than mock‐transfected cells (Figure 4A). The elevated portion of the uptake, which was ATP13A3‐specific, was markedly reduced under the ATP‐depleted condition (Figure 4A) and by the D498N mutation (Figure 4B), indicating ATP‐dependent transport directly coupled with ATP hydrolysis for ATP13A3‐mediated spermidine transport.

**FIGURE 4:**
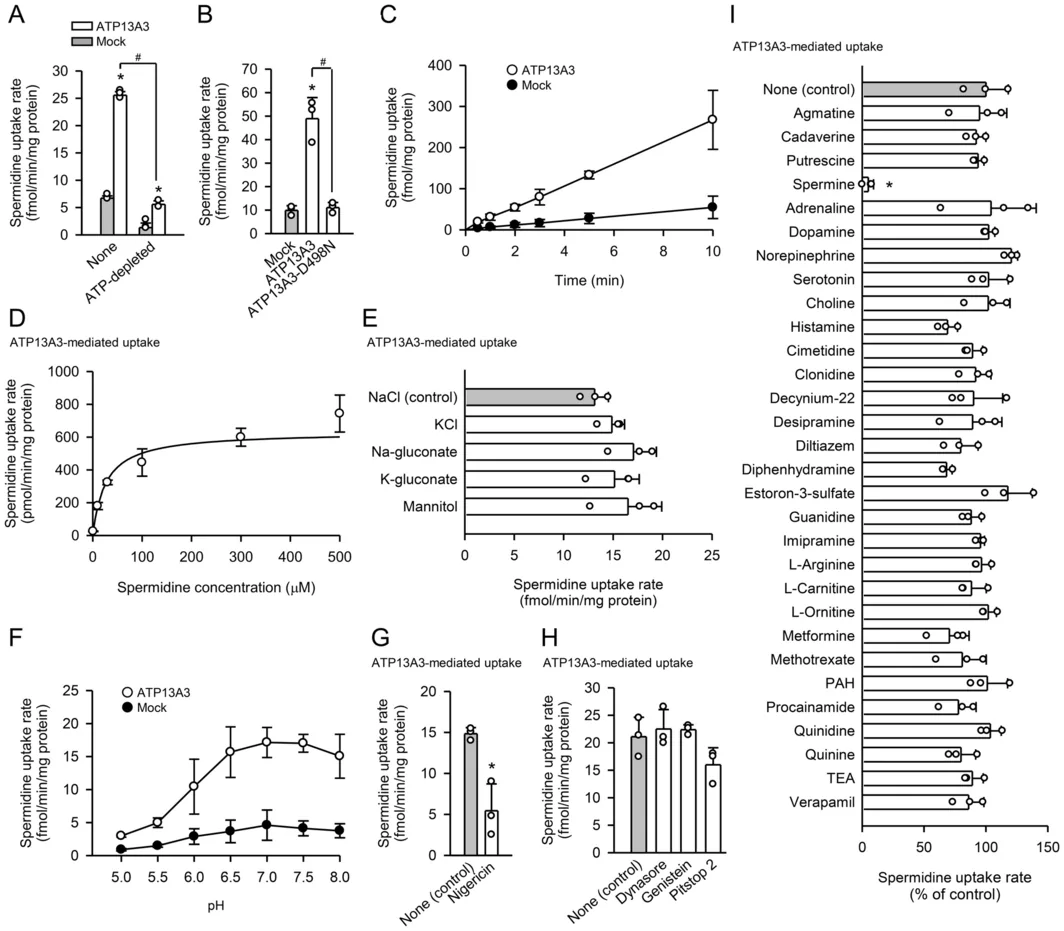
Functional characteristics of ATP13A3‐mediated spermidine transport in transiently transfected COS‐7 cells. (A) Effect of ATP depletion. The uptake of [^3^H]spermidine (1 nM) was measured for 5 min at pH 7.4 and 37°C in ATP13A3‐transfected cells and in mock‐transfected cells with or without ATP depletion. ATP depletion was induced by preincubation (20 min) in the presence of sodium azide (10 mM), sodium fluoride (10 mM), and 3‐O‐methyl‐D‐glucopyranose (25 mM). (B) Effect of D498N mutation in the ATPase domain. The uptake of [^3^H]spermidine (1 nM) was measured for 5 min at pH 7.4 and 37°C in cells transfected with the WT or D498N mutant of FLAG‐tagged ATP13A3 and in mock‐transfected cells. (C) Time course of spermidine uptake. The uptake of [^3^H]spermidine (1 nM) was measured at pH 7.4 and 37°C in ATP13A3‐transfected cells and in mock‐transfected cells. (D) Concentration dependence. The specific uptake of [^3^H]spermidine by ATP13A3 in its transfected cells was evaluated for 5 min at pH 7.4 and 37°C. The *V*_max_ and *K*_m_ were 632 ± 97 pmol/min/mg protein and 25.9 ± 7.5 μM, respectively. (E) Effect of ionic composition. The specific uptake of [^3^H]spermidine (1 nM) by ATP13A3 in its transfected cells was evaluated for 5 min at pH 7.4 and 37°C, where NaCl in the control medium was replaced as indicated. (F) Effect of extracellular pH. The uptake of [^3^H]spermidine (1 nM) was measured for 5 min at 37°C in ATP13A3‐transfected cells and in mock‐transfected cells. (G) Effect of intracellular acidification. The specific uptake of [^3^H]spermidine (1 nM) by ATP13A3 in its transfected cells was evaluated for 5 min at pH 7.4 and 37°C after pretreatment with nigericin (10 μM) for 5 min to induce intracellular acidification. (H) Effect of endocytosis inhibitors. The specific uptake of [^3^H]spermidine (1 nM) by ATP13A3 in its transfected cells was evaluated for 5 min at pH 7.4 and 37°C after pretreatment with inhibitors (50 μM for pitstop 2 and 100 μM for the others) for 30 min. (I) Effect of various compounds. The specific uptake of [^3^H]spermidine (1 nM) by ATP13A3 in its transfected cells was evaluated for 5 min at pH 7.4 and 37°C in the presence or absence of test compounds (50 μM for decynium‐22 and 100 μM for others). PAH, *p*‐aminohippuric acid; TEA, tetraethylammonium. Data are presented as means ± standard deviations from three biological replicates (*N* = 3), each of which was performed in duplicate (*n* = 2) as technical replication. Statistical significance was determined using analysis of variance followed by Bonferroni's test (A, B) or Dunnett's test (E, H, I), or using Student's *t*‐test (G). *p < 0.05 vs. the control or mock group. #*p* < 0.05 vs. the indicated group.

Based on the time course of spermidine uptake increasing in proportion to time up to 10 min in ATP13A3‐transfected cells, with minimal uptake in mock cells (Figure 4C), a 5‐min period was selected for uptake evaluations in subsequent functional analyses. Kinetic analysis demonstrated saturable ATP13A3‐mediated spermidine transport with a *V*_max_ of 632 pmol/min/mg protein and a *K*_m_ of 25.9 μM (Figure 4D). These parameters indicate a higher affinity but a lower capacity for spermidine than for putrescine. KCl, Na‐gluconate, K‐gluconate, or mannitol had no effect on ATP13A3‐specific spermidine uptake (Figure 4E), suggesting that, similar to putrescine transport, spermidine transport is not influenced by Na^+^, K^+^, or Cl^−^. Spermidine uptake was increased with an increase in pH only in ATP13A3‐transfected cells (Figure 4F) and ATP13A3‐specific spermidine uptake was significantly reduced by nigericin pretreatment (Figure 4G), indicating a pH‐sensitive characteristic but not H^+^/spermidine exchange as a mechanism. Furthermore, none of the endocytosis inhibitors altered ATP13A3‐specific spermidine uptake (Figure 4H), suggesting that endocytosis is not involved in that.

However, the profile of the effect of various compounds on the ATP13A3‐specific uptake of spermidine (1 nM) was quite different from the profile observed for ATP13A‐specific putrescine uptake (Figure 4I), exhibiting a significant inhibition only by spermine. This result suggests the weaker interaction of potential inhibitors with ATP13A3 in spermidine transport than in putrescine transport. The greater IC_50_ values of agmatine and cadaverine for the former than for the latter were in accordance with the suggestion (Table 1). The IC_50_ of putrescine (262 μM) for spermidine transport was also greater than its *K*_m_ (62.8 μM), suggesting its weaker interaction with ATP13A3 in the inhibition than in transport, whereas the IC_50_ of spermidine for putrescine transport (21.3 μM) was comparable to its *K*_m_ (25.9 μM), suggesting its interaction with ATP13A3 at comparable levels of affinity in the inhibition and transport, and the IC_50_ values of spermine were comparable for putrescine transport and for spermidine transport, suggesting its interaction with ATP13A3 at comparable levels of affinity in the inhibition for both.

### 3.5 Characterization of ATP13A4‐Mediated Putrescine Transport in Transiently Transfected COS‐7 Cells

For the detailed functional characterization of ATP13A4 as a putrescine transporter, uptake assays were performed in COS‐7 cells transiently transfected with ATP13A4. The uptake of [^3^H]putrescine (1 nM) was significantly elevated in ATP13A4‐transfected cells relative to mock control cells at pH 7.4, and the elevated portion of the uptake, which was ATP13A4‐specific one, was markedly reduced under the ATP‐depleted condition (Figure 5A) and in the D486N mutant (Figure 5B), which has previously been reported as a catalytically inactive/transport‐deficient mutant due to the mutation of the conserved phosphorylatable aspartate residue in the P domain of P‐type ATPase [35]. These results indicate ATP‐dependent transport involving an intact ATPase catalytic cycle for ATP13A4‐mediated putrescine transport, similar to ATP13A3‐mediated putrescine transport.

**FIGURE 5:**
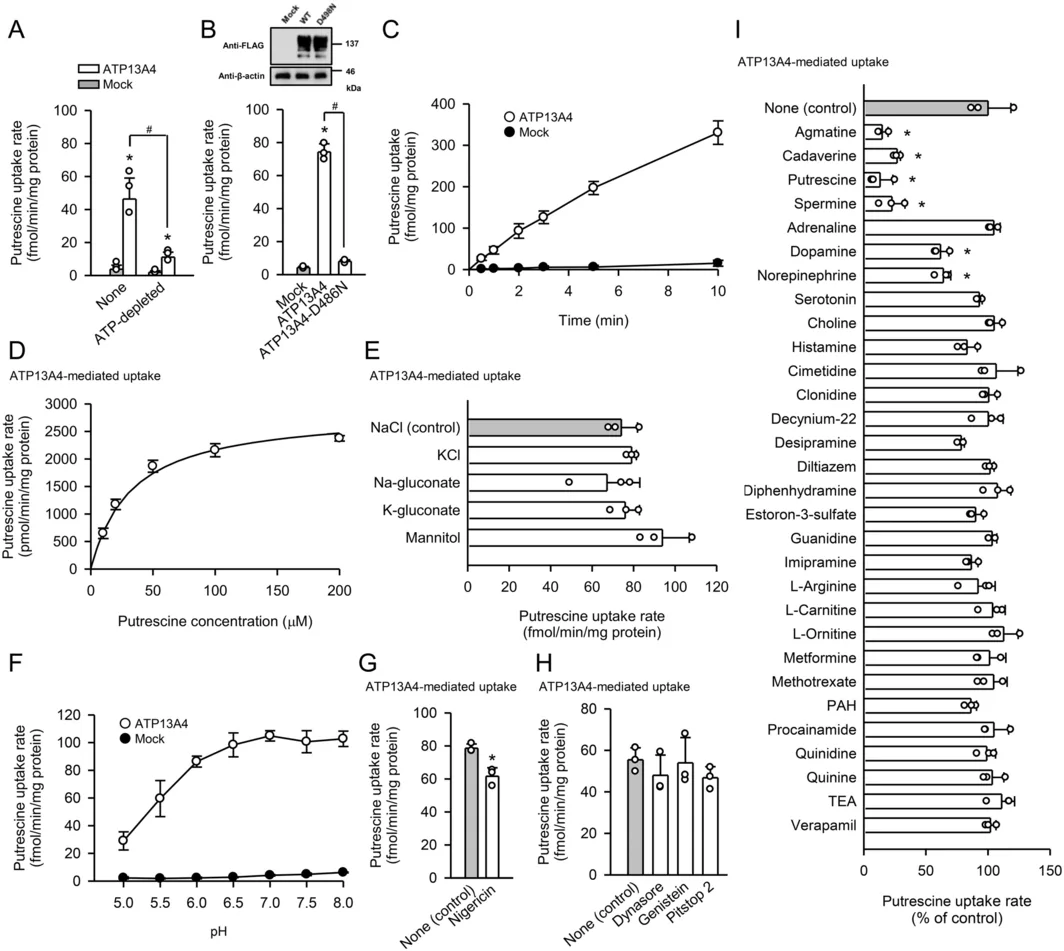
Functional characteristics of ATP13A4‐mediated putrescine transport in transiently transfected COS‐7 cells. (A) Effect of ATP depletion. The uptake of [^3^H]putrescine (1 nM) was measured for 3 min at pH 7.4 and 37°C in ATP13A4‐transfected cells and in mock‐transfected cells with or without ATP depletion. ATP depletion was induced by preincubation (20 min) in the presence of sodium azide (10 mM), sodium fluoride (10 mM), and 3‐O‐methyl‐D‐glucopyranose (25 mM). (B) Effect of D486N mutation in the ATPase domain. Expression of the WT and D486N mutant of FLAG‐tagged ATP13A4 in cells transfected with them was confirmed by Western blotting using an anti‐FLAG antibody, with β‐Actin serving as a loading control (upper panel). The uptake of [^3^H]putrescine (1 nM) was measured for 3 min at pH 7.4 and 37°C in cells transfected with the WT or D486N mutant of FLAG‐tagged ATP13A4 and in mock‐transfected cells (lower panel). (C) Time course of putrescine uptake. The uptake of [^3^H]putrescine (1 nM) was measured at pH 7.4 and 37°C in ATP13A4‐transfected cells and in mock‐transfected cells. (D) Concentration dependence. The specific uptake of [^3^H]putrescine by ATP13A4 in its transfected cells was evaluated for 3 min at pH 7.4 and 37°C. The *V*_max_ and *K*_m_ were 2870 ± 230 pmol/min/mg protein and 32.5 ± 9.1 μM, respectively. (E) Effect of ionic composition. The specific uptake of [^3^H]putrescine (1 nM) by ATP13A4 in its transfected cells was evaluated for 3 min at pH 7.4 and 37°C, where NaCl in the control medium was replaced as indicated. (F) Effect of extracellular pH. The uptake of [^3^H]putrescine (1 nM) was measured for 3 min at 37°C in ATP13A4‐transfected cells and in mock‐transfected cells. (G) Effect of intracellular acidification. The specific uptake of [^3^H]putrescine (1 nM) by ATP13A4 in its transfected cells was evaluated for 3 min at pH 7.4 and 37°C after pretreatment with nigericin (10 μM) for 5 min to induce intracellular acidification. (H) Effect of endocytosis inhibitors. The specific uptake of [^3^H]putrescine (1 nM) by ATP13A4 in its transfected cells was evaluated for 3 min at pH 7.4 and 37°C after pretreatment with inhibitors (50 μM for pitstop 2 and 100 μM for the others) for 30 min. (I) Effect of various compounds. The specific uptake of [^3^H]putrescine (1 nM) by ATP13A4 in its transfected cells was evaluated for 3 min at pH 7.4 and 37°C in the presence or absence of test compounds (50 μM for decynium‐22 and 100 μM for others). PAH, *p*‐aminohippuric acid; TEA, tetraethylammonium. Data are presented as means ± standard deviations from three biological replicates (*N* = 3), each of which was performed in duplicate (*n* = 2) as technical replication. Statistical significance was determined using analysis of variance followed by Bonferroni's test (A, B) or Dunnett's test (E, H, I), or using Student's *t*‐test (G). *p < 0.05 vs. the control or mock group. #*p* < 0.05 vs. the indicated group.

Putrescine uptake increased in proportion to time in ATP13A4‐transfected cells, whereas it was minimal in mock cells during the initial 3‐min period (Figure 5C) and, accordingly, this period was used for uptake evaluations in subsequent functional analyses. Saturable putrescine transport by ATP13A4 was observed and kinetically characterized with a *V*_max_ of 2870 pmol/min/mg protein and a *K*_m_ of 32.5 μM (Figure 5D). Compared to ATP13A3, ATP13A4 had a similar capacity but a higher affinity for putrescine. KCl, Na‐gluconate, K‐gluconate, or mannitol had no effect on ATP13A4‐specific putrescine uptake (Figure 5E), suggesting that ATP13A4‐mediated putrescine transport is not influenced by Na^+^, K^+^, or Cl^−^, similar to ATP13A3‐mediated one. Putrescine uptake was increased with an increase in pH only in ATP13A4‐transfected cells (Figure 5F), and ATP13A4‐specific putrescine uptake was significantly reduced by pretreatment with nigericin (Figure 5G), indicating a pH‐sensitive characteristic but not H^+^/putrescine exchange as a mechanism. Furthermore, none of the endocytosis inhibitors altered ATP13A4‐specific putrescine uptake (Figure 5H), suggesting that endocytosis is not involved in that.

The profile of the effect of various compounds on the specific uptake of putrescine (1 nM) by ATP13A4 is shown in Figure 5I. The specific uptake was strongly inhibited by polyamines (spermidine and spermine) and polyamine analogs (agmatine and cadaverine) and was moderately inhibited by dopamine and norepinephrine, which are bioactive amines, manifesting differences in the inhibition profile compared with that for ATP13A3‐mediated putrescine transport. The IC_50_ values of polyamines and polyamine analogs are shown in Table 1. Whereas the IC_50_ of spermine for ATP13A4‐mediated putrescine transport was comparable to that for ATP13A3‐mediated one, suggesting its interaction with ATP13A3 and ATP13A4 at comparable levels of affinity in the inhibition, the IC_50_ values of the other compounds for ATP13A4‐mediated one were smaller than their respective IC_50_ values for ATP13A3‐mediated one, suggesting higher affinity in the inhibition of ATP13A4.

### 3.6 Characterization of ATP13A4‐Mediated Spermidine Transport in Transiently Transfected COS‐7 Cells

The spermidine transport function of ATP13A4 was also examined in COS‐7 cells transiently transfected with ATP13A4. The uptake of [^3^H]spermidine (1 nM) was significantly higher in ATP13A4‐transfected cells than in mock control cells at pH 7.4, and the elevated portion of the uptake was markedly reduced under the ATP‐depleted condition (Figure 6A) and by the D486N mutation (Figure 6B), indicating ATP‐dependent transport directly coupled with ATP hydrolysis for ATP13A4‐mediated spermidine transport.

**FIGURE 6:**
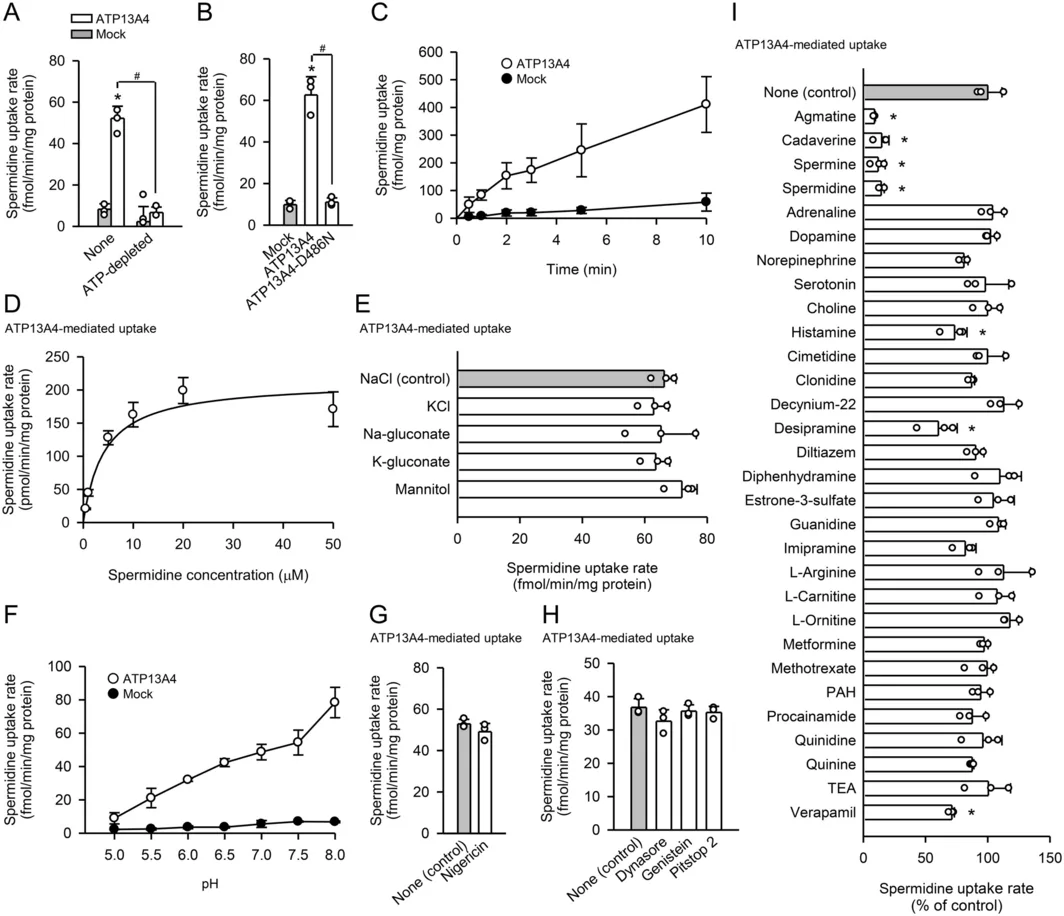
Functional characteristics of ATP13A4‐mediated spermidine transport in transiently transfected COS‐7 cells. (A) Effect of ATP depletion. The uptake of [^3^H]spermidine (1 nM) was measured for 2 min at pH 7.4 and 37°C in ATP13A4‐transfected cells and in mock‐transfected cells with or without ATP depletion. ATP depletion was induced by preincubation (20 min) in the presence of sodium azide (10 mM), sodium fluoride (10 mM), and 3‐O‐methyl‐D‐glucopyranose (25 mM). (B) Effect of D486N mutation in the ATPase domain. The uptake of [^3^H]spermidine (1 nM) was measured for 2 min at pH 7.4 and 37°C in cells transfected with the WT or D486N mutant of FLAG‐tagged ATP13A4 and in mock‐transfected cells. (C) Time course of spermidine uptake. The uptake of [^3^H]spermidine (1 nM) was measured at pH 7.4 and 37°C in ATP13A4‐transfected cells and in mock‐transfected cells. (D) Concentration dependence. The specific uptake of [^3^H]spermidine by ATP13A4 in its transfected cells was evaluated for 2 min at pH 7.4 and 37°C. The *V*_max_ and *K*_m_ were 212 ± 25 pmol/min/mg protein and 4.17 ± 0.46 μM, respectively. (E) Effect of ionic composition. The specific uptake of [^3^H]spermidine (1 nM) by ATP13A4 in its transfected cells was evaluated for 2 min at pH 7.4 and 37°C, where NaCl in the control medium was replaced as indicated. (F) Effect of extracellular pH. The uptake of [^3^H]spermidine (1 nM) was measured for 2 min at 37°C in ATP13A4‐transfected cells and in mock‐transfected cells. (G) Effect of intracellular acidification. The specific uptake of [^3^H]spermidine (1 nM) by ATP13A4 in its transfected cells was evaluated at pH 7.4 and 37°C after pretreatment with nigericin (10 μM) for 5 min to induce intracellular acidification. (H) Effect of endocytosis inhibitors. The specific uptake of [^3^H]spermidine (1 nM) by ATP13A4 in its transfected cells was evaluated for 2 min at pH 7.4 and 37°C after pretreatment with inhibitors (50 μM for pitstop 2 and 100 μM for the others) for 30 min. (I) Effect of various compounds. The specific uptake of [^3^H]spermidine (1 nM) by ATP13A4 in its transfected cells was evaluated for 2 min at pH 7.4 and 37°C in the presence or absence of test compounds (50 μM for decynium‐22 and 100 μM for the others). PAH, *p*‐aminohippuric acid; TEA, tetraethylammonium. Data are presented as means ± standard deviations from three biological replicates (*N* = 3), each of which was performed in duplicate (*n* = 2) as technical replication. Statistical significance was determined using analysis of variance followed by Bonferroni's test (A, B) or Dunnett's test (E, H, I), or using Student's *t*‐test (G). *p < 0.05 vs. the control or mock group. #*p* < 0.05 vs. the indicated group.

Spermidine uptake increased in proportion to time up to 2 min in ATP13A4‐transfected cells, with minimal uptake in mock cells (Figure 6C). Accordingly, a 2‐min period was used for uptake evaluations in subsequent functional analyses. Saturable spermidine transport by ATP13A4 was observed and kinetically characterized with a *V*_max_ of 212 pmol/min/mg protein and a *K*_m_ of 4.17 μM (Figure 6D). These parameters indicate a higher affinity but a lower capacity for spermidine than for putrescine in ATP13A4‐mediated transport, similar to ATP13A3‐mediated transport, and a higher affinity but a lower capacity for spermidine in ATP13A4‐mediated transport than in ATP13A3‐mediated transport. The *K*_m_ was comparable to the IC_50_ of spermidine for ATP13A4‐mediated putrescine transport. KCl, Na‐gluconate, K‐gluconate, or mannitol had no effect on ATP13A4‐specific spermidine uptake (Figure 6E), suggesting no influence of Na^+^, K^+^, or Cl^−^ on spermidine transport, similar to putrescine transport. Spermidine uptake was increased with an increase in pH only in ATP13A4‐transfected cells (Figure 6F) and ATP13A4‐specific spermidine uptake was not affected by nigericin pretreatment (Figure 6G), indicating a pH‐sensitive characteristic but not H^+^/spermidine exchange as a mechanism. Furthermore, none of the endocytosis inhibitors altered ATP13A4‐specific spermidine uptake (Figure 6H), suggesting that endocytosis is not involved in that.

The ATP13A4‐specific uptake of spermidine (1 nM) was strongly inhibited by polyamines (putrescine and spermine) and polyamine analogs (agmatine and cadaverine), and was moderately inhibited by histamine, desipramine, and verapamil (Figure 6I), manifesting differences in the inhibition profile compared with those for ATP13A4‐mediated putrescine transport and ATP13A3‐mediated spermidine transport. The IC_50_ values of polyamines and polyamine analogs are shown in Table 1. The IC_50_ of putrescine for ATP13A4‐mediated spermidine transport was smaller than its *K*_m_ for ATP13A4, suggesting higher affinity in its interaction with ATP13A4 in the inhibition of spermidine transport than in transport. The IC_50_ of spermine for ATP13A4‐mediated spermidine transport was comparable to those for ATP13A3‐mediated putrescine transport, ATP13A3‐mediated spermidine transport, and ATP13A4‐mediated putrescine transport, suggesting its interaction with ATP13A3 and ATP13A4 at comparable levels of affinity. The IC_50_ of agmatine for ATP13A4‐mediated spermidine transport was comparable to that for ATP13A4‐mediated putrescine transport, suggesting its interaction with ATP13A4 at comparable levels of affinity in the inhibition for both, but smaller than that for ATP13A3‐mediated spermidine transport, suggesting its higher affinity for ATP13A4. The IC_50_ of cadaverine for ATP13A4‐mediated spermidine transport was smaller than those for ATP13A4‐mediated putrescine transport and ATP13A3‐mediated spermidine transport, suggesting its higher affinity for ATP13A4 particularly in the inhibition of spermidine transport.

### 3.7 Tissue Distribution of ATP13A3 and ATP13A4 mRNAs

Quantitative real‐time PCR analysis using a standard curve‐based absolute quantification method revealed that the expression patterns of ATP13A3 and ATP13A4 mRNAs were distinct across human tissues. ATP13A3 mRNA was expressed in most tissues (Figure 7A), suggesting a general role for ATP13A3 in regulating polyamine homeostasis and associated cellular functions throughout the body. In contrast, ATP13A4 mRNA exhibited a more restricted expression pattern than ATP13A3 mRNA. It was expressed in several limited tissues, including the brain, lung, small intestine, colon, pancreas, and skin as the major ones (Figure 7B), suggesting its specialized roles in polyamine regulation in such tissues.

**FIGURE 7:**
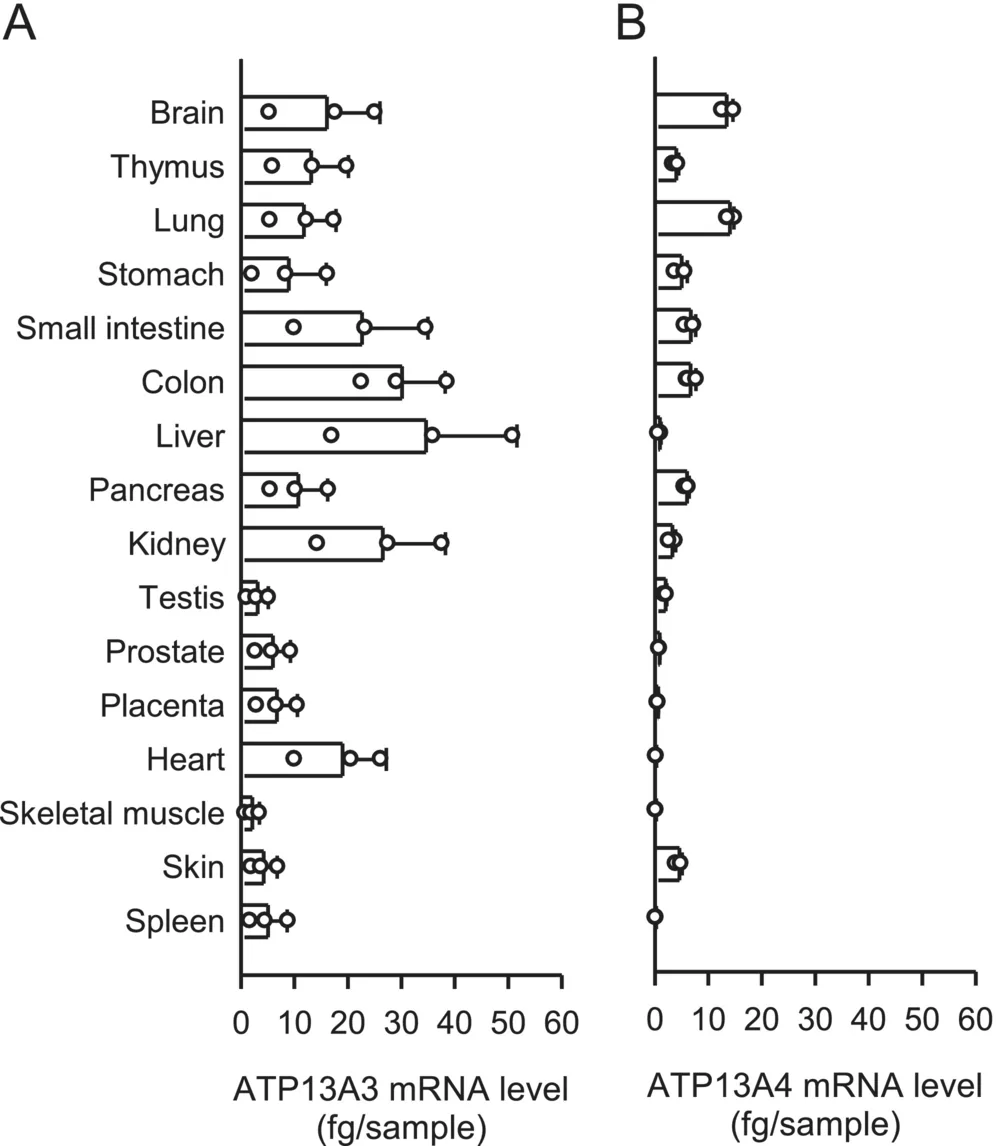
Expression of ATP13A3 and ATP13A4 in human tissues. The mRNA expression levels of ATP13A3 (A) and ATP13A4 (B) were quantified by real‐time PCR. The expression levels were normalized to those of ubiquitin C and calibrated using a standard curve to determine the absolute level in the total RNA sample from each tissue. Data are presented as means ± standard deviations. Assays were performed in triplicate (*n* = 3) as technical replication and repeated independently using three different samples (*N* = 3) prepared from the single total RNA sample from each tissue, which was from a single donor, for the calculation of data for presentation. Donor information for each total RNA sample is provided in Table S1.

### 3.8 Expression Profiles of ATP13A3 and ATP13A4 mRNAs in Human Cell Lines

We also performed quantitative real‐time PCR analysis in human cell lines to determine the expression profiles of ATP13A3 and ATP13A4 mRNAs. ATP13A3 exhibited ubiquitous expression in various cell lines originating from various organs (Figure 8A), which is consistent with its broad tissue distribution and supports the role of ATP13A3 as a housekeeping polyamine transporter essential for maintaining intracellular polyamine homeostasis across a wide range of cell types. The consistently robust expression in several cell types underscores the functional importance of ATP13A3 in fundamental cellular processes, irrespective of tissue origin or differentiation status. In contrast, ATP13A4 exhibited a more restricted expression pattern across the cell lines, being particularly abundant in HepG2 cells and detectable only in a few other cell lines (Figure 8B). The low or undetectable expression of ATP13A4 in many cell lines reinforces the notion that ATP13A4 is subject to strict transcriptional regulation and likely contributes to specialized, rather than ubiquitous, functions.

**FIGURE 8:**
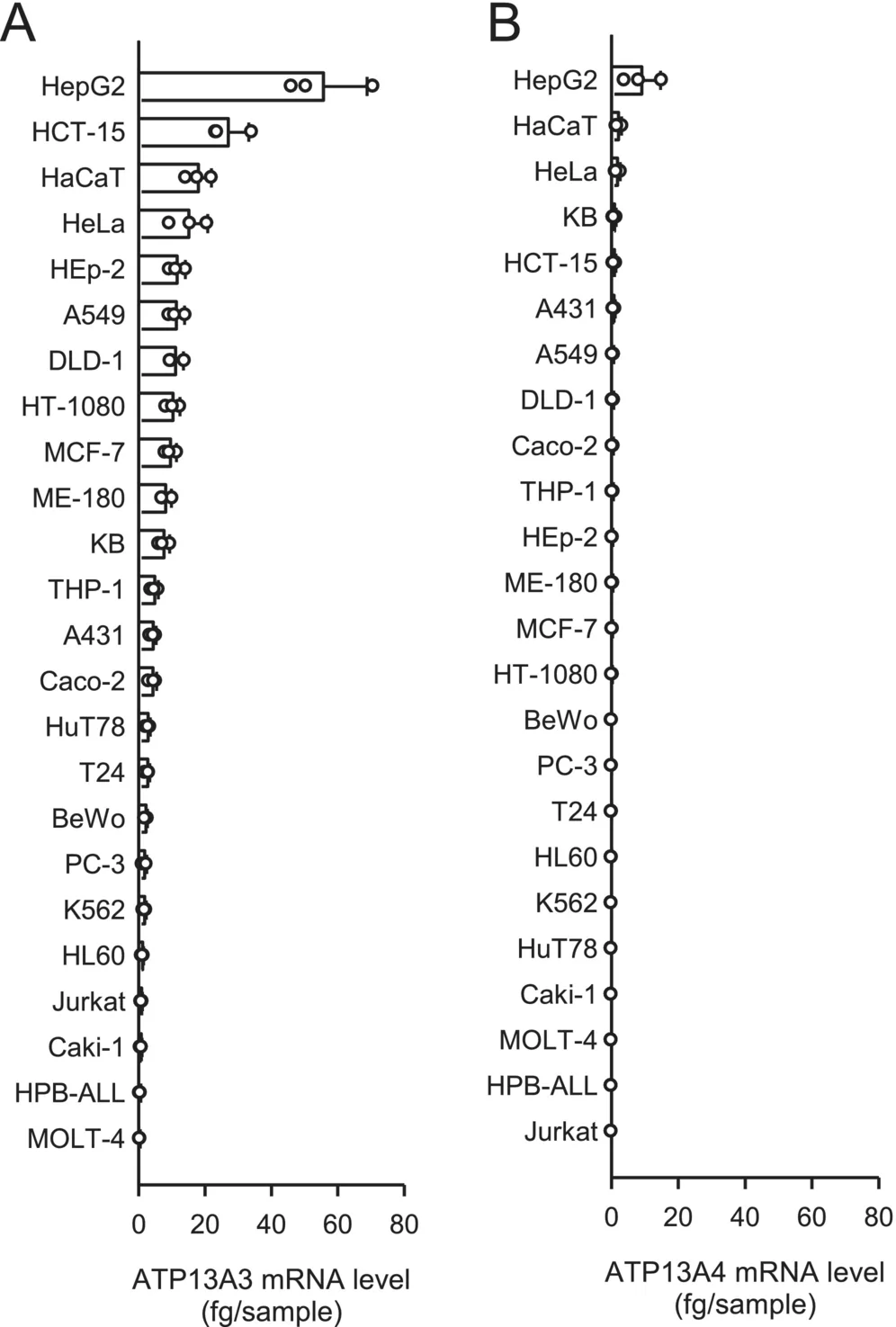
Expression of ATP13A3 and ATP13A4 in human cell lines. The mRNA expression levels of ATP13A3 (A) and ATP13A4 (B) were quantified by real‐time PCR. The expression levels were normalized to those of GAPDH and calibrated using a standard curve to determine the absolute level in each cell line. Data are presented as means ± standard deviations. Assays were performed in triplicate (*n* = 3) as technical replication and repeated independently using three different cell preparations (*N* = 3) for biological replication.

### 3.9 Role of ATP13A3 and ATP13A4 in Polyamine Uptake

The roles of ATP13A3 and ATP13A4 in polyamine uptake in human HepG2 and A549 cells were evaluated by RNA interference experiments. HepG2 cells were selected as a hepatic cell line with high expression levels of both ATP13A3 and ATP13A4. A549 cells were selected as a cell line with appreciable ATP13A3 expression, but with only minimal ATP13A4 expression. ATP13A3 and ATP13A4 knockdown using specific siRNAs significantly reduced the uptake of [^3^H]putrescine (1 nM) and, accordingly, their mRNA levels in HepG2 cells (Figure 9A–D), confirming the role of ATP13A3 and ATP13A4 in polyamine uptake. Similar results were observed for ATP13A3 in A549 cells (Figure 9E,F), demonstrating its role in polyamine uptake. In contrast, ATP13A4 knockdown had no detectable effects on putrescine uptake and ATP13A4 mRNA level in A549 cells (Figure 9G,H). It is likely because of low knockdown efficiency associated with inherently low expression (Figure 8B). Notably, two independent siRNAs targeting each transporter brought about consistent effects on putrescine uptake and mRNA expression in each set of experiments. Therefore, the observed effects are least likely attributable to siRNA‐specific off‐target effects. The consistent effects observed with ATP13A3 knockdown in both HepG2 and A549 cells, together with those observed with ATP13A4 knockdown in HepG2 cells, further support the contribution of endogenous ATP13A3 and ATP13A4 to cellular polyamine uptake.

**FIGURE 9:**
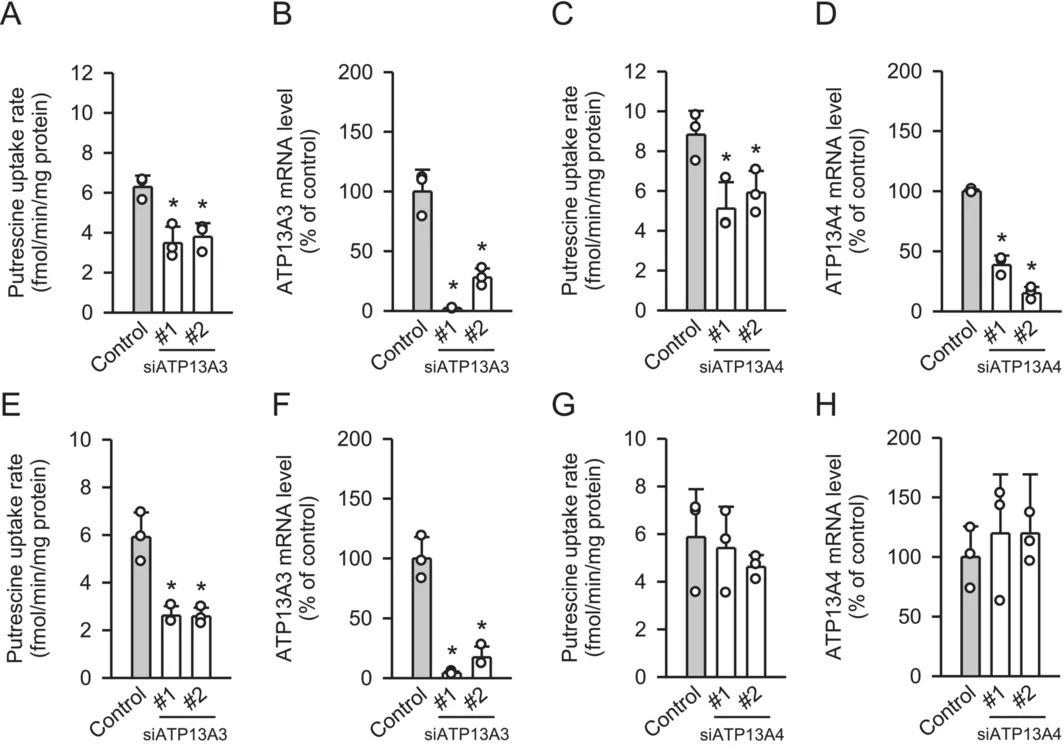
Effect of the knockdown of ATP13A3 and ATP13A4 on putrescine uptake in human cell lines. (A) The uptake of [^3^H]putrescine (1 nM) was measured for 5 min at pH 7.4 and 37°C in HepG2 cells transfected with the siRNAs targeting ATP13A3 mRNA. (B) The levels of ATP13A3 mRNA were determined by real‐time PCR and normalized to those of GAPDH mRNA. (C) The uptake of [^3^H]putrescine (1 nM) was measured for 5 min at pH 7.4 and 37°C in HepG2 cells transfected with the siRNAs targeting ATP13A4 mRNA. (D) The levels of ATP13A4 mRNA were determined by real‐time PCR and normalized to those of GAPDH mRNA. (E) The uptake of [^3^H]putrescine (1 nM) was measured for 5 min at pH 7.4 and 37°C in A549 cells transfected with the siRNAs targeting ATP13A3 mRNA. (F) The levels of ATP13A3 mRNA were determined by real‐time PCR and normalized to those of GAPDH mRNA. (G) The uptake of [^3^H]putrescine (1 nM) was measured for 5 min at pH 7.4 and 37°C in A549 cells transfected with the siRNAs targeting ATP13A4 mRNA. (H) The levels of ATP13A4 mRNA were determined by real‐time PCR and normalized to those of GAPDH mRNA. Data are presented as the means ± standard deviations of three biological replicates (*N* = 3), each of which was performed in duplicate (*n* = 2) as technical replication. Statistical significance was determined using analysis of variance followed by Dunnett's test. *p < 0.05 vs. the control group.

Because intracellular polyamine depletion has been reported to enhance cellular polyamine uptake, with a more pronounced increase in putrescine uptake than in spermidine uptake [36], we next examined the effects of polyamine depletion induced by 2‐difluoromethylornithine (DFMO), an inhibitor of polyamine biosynthesis. In HepG2 cells, DFMO treatment significantly increased putrescine uptake (Figure S1A) and also spermidine uptake (Figure S1B), although to a lesser extent than putrescine uptake. Similar trends were observed in A549 cells, although the DFMO‐induced increase was significant in putrescine uptake (Figure S1C) but not in spermidine uptake (Figure S1D). Thus, polyamine depletion by DFMO was demonstrated to enhance polyamine uptake, with a more pronounced effect on putrescine uptake, as reported previously [37]. However, DFMO treatment altered neither ATP13A3 nor ATP13A4 mRNA expression in both HepG2 cells (Figure S1E,F) and A549 cells (Figure S1G,H), indicating that the DFMO‐induced enhancement of polyamine uptake was not accompanied by transcriptional upregulation of ATP13A3 or ATP13A4. This adaptive response may involve regulatory mechanisms other than transcriptional upregulation of these transporters.

## 4 Discussion

This study establishes ATP13A3 and ATP13A4 as ATP‐dependent polyamine transporters localized to the plasma membrane, thereby expanding the functional framework of the P5B‐type subclass within the P‐type ATPase superfamily. Generally, P‐type ATPases have been known as transporters of inorganic ions, including sodium, potassium, calcium, hydrogen, and copper, as well as lipid substrates such as phospholipids. This study is the first to show that ATP13A3 and ATP13A4 mediate the ATP‐dependent uptake of small hydrophilic organic cations—specifically the polyamines putrescine and spermidine—across the plasma membrane.

Previous evidence indicating that ATP13A3 and ATP13A4 function within intracellular compartments, particularly late endosomes and lysosomes, is largely based on co‐localization of organellar markers and BODIPY‐conjugated fluorescent polyamine analogs. However, such compounds are susceptible to lysosomal entrapment due to their physicochemical properties, including lipophilicity and the presence of weakly basic functional groups [38, 39, 40]. These compounds undergo protonation under acidic conditions, leading to accumulation in the protonated form, after diffusive permeation across the lysosomal membrane, independent of transporter activity. Consequently, these observations risk confusing physicochemical sequestration with transporter‐mediated active accumulation. In contrast, these limitations were overcome by combining immunofluorescence staining and uptake assays using radiolabeled polyamines in the present study. The results demonstrate that ATP13A3 and ATP13A4 are localized to the plasma membrane and function as active polyamine transporters.

The present findings provide the molecular basis for the ATP‐dependent uptake of polyamines across various mammalian cell types. Specifically, ATP13A3 exhibited the *K*_m_ values of 62.8 μM for putrescine (Figure 3D) and 25.9 μM for spermidine (Figure 4D), while ATP13A4 exhibited the *K*_m_ values of 32.5 μM for putrescine (Figure 5D) and 4.17 μM for spermidine (Figure 6D). Notably, previously reported *K*_m_ values for the uptake of putrescine and spermidine in various human cell lines fall within a similar range to those of ATP13A3 and ATP13A4, supporting their roles as major contributors to constitutive polyamine import. For instance, in HL‐60 cells, the *K*_m_ values are reportedly 52 μM and 7.9 μM, respectively, for the uptake of putrescine and spermidine [41]. In addition, ATP13A3 was ubiquitously expressed across a wide range of tissues (Figure 7A) and cell lines (Figure 8A), whereas ATP13A4 exhibited a more restricted expression pattern in both tissues (Figure 7B) and cell lines (Figure 8B). These profiles suggest distinct physiological roles, with ATP13A3 likely functioning as a major polyamine importer widely present throughout the body, whereas ATP13A4 functioning for specialized roles in limited tissues and cell types.

ATP13A3 and ATP13A4 share similarities with ATP13A2, a lysosomal polyamine exporter linked to early‐onset Parkinson's disease, in their function as polyamine transporters. Collectively, these P5B‐type ATPases constitute a subfamily implicated in polyamine transport across distinct biological membranes: lysosomal membrane for ATP13A2 and plasma membrane for ATP13A3 and ATP13A4. This organization underscores the necessity for compartmentalized polyamine regulation and implies that perturbation at any level could result in tissue‐specific pathologies, compromising cellular homeostasis and predisposing the affected tissues to degeneration or maladaptive remodeling.

From a pathophysiological perspective, ATP13A3 has been genetically implicated in pulmonary arterial hypertension, which is characterized by endothelial hyperplasia and vascular remodeling. Given the role of polyamines in cellular proliferation and metabolic adaptation, impaired ATP13A3‐mediated polyamine uptake may contribute to such endothelial dysfunction and disease progression. ATP13A4 is associated with neurodevelopmental disorders, including autism spectrum disorder and intellectual disabilities. The expression of ATP13A4 in the brain observed in the present study is consistent with the notion that impaired ATP13A4‐mediated polyamine handling may affect neural development and function. Several mutations in ATP13A4 may lead to loss of its function and result in defective polyamine handling during neural development, which may underlie key aspects of disease etiology. Identifying ATP13A3 and ATP13A4 as transporters involved in the ATP‐dependent cellular uptake of polyamines provides a molecular basis for exploring potential therapeutic strategies targeting polyamine dysregulation by transporter‐specific interventions.

In conclusion, this study redefines the substrate specificity and localization profile of the P‐type ATPase superfamily by characterizing ATP13A3 and ATP13A4 as ATP‐driven importers of polyamines at the plasma membrane. Their functional properties, tissue distribution, and roles in diseases underscore their physiological importance and pathological relevance. This study should help elucidate the networks regulating polyamine homeostasis and identify potential therapeutic targets for disorders characterized by changes in polyamine uptake.

## Author Contributions

All the authors have read and agreed to the published version of the manuscript. Haruka Morimoto and Mione Yamasaki performed the laboratory experiments, analyzed the data, and prepared the figures. Tomoya Yasujima conceived the idea, designed the experiments, performed the laboratory experiments, and prepared the figures and the original draft. Takahiro Yamashiro conceived the idea. Hiroaki Yuasa revised the manuscript and supervised the study.

## Funding

This study was supported in part by JSPS KAKENHI (Grant Number JP20K07159).

## Conflicts of Interest

The authors declare no conflicts of interest.

## Supporting information


**Table S1:** Materials and cell lines.
**Table S2:** Primers for cDNA amplification of ATP13A family transporters.
**Table S3:** Primers for the generation of the cDNAs for ATP13A3 and ATP13A4 mutants.
**Table S4:** Primers for FLAG‐tag insertion into the extracellular loop of ATP13A3 and ATP13A4.
**Table S5:** Primers for the real‐time PCR analyses of the expression of ATP13A3 and ATP13A4.
**Table S6:** Sequences of the siRNAs for ATP13A3 and ATP13A4.

**Figure S1:** Effects of DFMO treatment on polyamine uptake and ATP13A3 and ATP13A4 mRNA expression in HepG2 and A549 cells. (A) The uptake of [^3^H]putrescine (1 nM) was measured for 5 min at pH 7.4 and 37°C in HepG2 cells after incubation with or without DFMO (5 mM) for 48 h. (B) The uptake of [^3^H]spermidine (1 nM) was similarly evaluated in HepG2 cells. (C) The uptake of [^3^H]putrescine (1 nM) was similarly evaluated in A549 cells. (D) The uptake of [^3^H]spermidine (1 nM) was similarly evaluated in A549 cells. (E) The levels of ATP13A3 mRNA in HepG2 cells were quantified by real‐time PCR after the incubation with or without DFMO. (F) The levels of ATP13A4 mRNA in HepG2 cells were similarly evaluated. (G) The levels of ATP13A3 mRNA in A549 cells were similarly evaluated. (H) The levels of ATP13A4 mRNA in A549 cells were similarly evaluated. Data are presented as means ± standard deviations from three biological replicates (*N* = 3), each of which was performed in duplicate (*n* = 2) as technical replication. Statistical significance was determined using Student's *t*‐test. *p < 0.05 vs. the control group.

## Data Availability

The data that support the findings of this study are available on request from the corresponding.
