# Supplementary material for: Functional Characterization of the P‐Type ATPases ATP13A3 and ATP13A4 as Plasma Membrane Polyamine Transporters
Source: FASEB J. 2026 Oct 13;40(20):e72383. doi: 10.1096/fj.202603205RR (PMC13660768; doi:10.1096/fj.202603205RR)
Supplement: Supplementary file 1 [file FSB2-40-e72383-s001.docx]

**FASEB Journal**

Supporting information

Functional characterization of the P-type ATPases ATP13A3 and ATP13A4 as plasma membrane polyamine transporters

Haruka Morimoto, Tomoya Yasujima, Mione Yamasaki, Takahiro Yamashiro, Hiroaki Yuasa

Department of Biopharmaceutics, Graduate School of Pharmaceutical Sciences, Nagoya City University, 3-1 Tanabe-dori, Mizuho-ku, Nagoya 467-8603, Japan

Figure S1

FIGURE S1. Effects of DFMO treatment on polyamine uptake and ATP13A3 and ATP13A4 mRNA expression in HepG2 and A549 cells. (A) The uptake of [³H]putrescine (1 nM) was measured for 5 minutes at pH 7.4 and 37 °C in HepG2 cells after incubation with or without DFMO (5 mM) for 48 hours. (B) The uptake of [³H]spermidine (1 nM) was similarly evaluated in HepG2 cells. (C) The uptake of [³H]putrescine (1 nM) was similarly evaluated in A549 cells. (D) The uptake of [³H]spermidine (1 nM) was similarly evaluated in A549 cells. (E) The levels of ATP13A3 mRNA in HepG2 cells were quantified by real-time PCR after the incubation with or without DFMO. (F) The levels of ATP13A4 mRNA in HepG2 cells were similarly evaluated. (G) The levels of ATP13A3 mRNA in A549 cells were similarly evaluated. (H) The levels of ATP13A4 mRNA in A549 cells were similarly evaluated. Data are presented as means ± standard deviations from three biological replicates (N = 3), each of which was performed in duplicate (n = 2) as technical replication. Statistical significance was determined using Student’s t-test. **p* < 0.05 vs. the control group.
