# Supplementary material for: Functional Characterization of the P‐Type ATPases ATP13A3 and ATP13A4 as Plasma Membrane Polyamine Transporters
Source: FASEB J. 2026 Oct 13;40(20):e72383. doi: 10.1096/fj.202603205RR (PMC13660768; doi:10.1096/fj.202603205RR)
Supplement: Supplementary file 2 [file FSB2-40-e72383-s002.docx]

**FASEB Journal**

Supporting information

Functional characterization of the P-type ATPases ATP13A3 and ATP13A4 as plasma membrane polyamine transporters

Haruka Morimoto, Tomoya Yasujima, Mione Yamasaki, Takahiro Yamashiro, Hiroaki Yuasa

Department of Biopharmaceutics, Graduate School of Pharmaceutical Sciences, Nagoya City University, 3-1 Tanabe-dori, Mizuho-ku, Nagoya 467-8603, Japan

Table S1

Materials and cell lines

| Reagent or resource | Source | Identifer |
| --- | --- | --- |
| Antibodies |  |  |
| Anti-FLAG (1:1000) | FUJIFILM Wako | Cat#014-22383;  RRID: AB_10659717 |
| Anti-β-actin (1:1000) | Proteintech | Cat#60008-1-Ig;  RRID: AB_2289225 |
| Goat anti-mouse IgG secondary antibody,  HRP (1:10000) | Jackson ImmunoResearch | Cat#115-035-003;  RRID: AB_10015289 |
| Goat anti-mouse IgG secondary antibody,  Alexa Fluor 488 (1:500) | ThermoFisher | Cat#A11070;  RRID: AB_2534114 |
|  |  |  |
| Bacterials and virus strains |  |  |
| OmniMax 2 T1R | ThermoFisher | Cat#C854003 |
|  |  |  |
| Biological samples |  |  |
| Human small intestine total RNA | BioChain | Cat#R1234226  Donor information  Age: 26, Sex: male  Clinical diagnosis: normal |
| Human brain total RNA | BioChain | Cat#R1234035  Donor information  Age: 24, Sex: male  Clinical diagnosis: normal |
| Human colon total RNA | BioChain | Cat#R1234090  Donor information  Age: 29, Sex: male  Clinical diagnosis: normal |
| Human heart total RNA | BioChain | Cat#R1234122  Donor information  Age: 29, Sex: male  Clinical diagnosis: normal |
| Human kidney total RNA | BioChain | Cat#R1234142  Donor information  Age: 26, Sex: male  Clinical diagnosis: normal |
| Human liver total RNA | BioChain | Cat#R1234149  Donor information  Age: 51, Sex: male  Clinical diagnosis: normal |
| Human lung total RNA | BioChain | Cat#R1234152  Donor information  Age: 71, Sex: male  Clinical diagnosis: normal |
| Human pancreas total RNA | BioChain | Cat#R1234188  Donor information  Age: 44, Sex: male  Clinical diagnosis: normal |
| Human placenta total RNA | BioChain | Cat#R1234200  Donor information  Not available. |
| Human prostate RNA | BioChain | Cat#R1234201  Donor information  Not available. |
| Human skeletal muscle RNA | BioChain | Cat#R1234171  Donor information  Age: 30, Sex: female  Clinical diagnosis: normal |
| Human skin total RNA | BioChain | Cat#R1234218  Donor information  Not available. |
| Human spleen total RNA | BioChain | Cat#R1234246  Donor information  Age: 30, Sex: male  Clinical diagnosis: normal |
| Human stomach total RNA | BioChain | Cat#R1234248  Donor information  Age: 24, Sex: male  Clinical diagnosis: normal |
| Human testis total RNA | BioChain | Cat#R1234260  Donor information  Not available. |
| Human thymus total RNA | BioChain | Cat#R1234264  Donor information  Age: 27, Sex: male  Clinical diagnosis: normal |
|  |  |  |
| Chemicals, peptides, and recombinant proteins | |  |
| DMEM | FUJIFILM Wako | Cat#043-30085 |
| Opti-MEM | Thermo | Cat#31985070 |
| FBS | Sigma-Aldrich | Cat#173012 |
| Penicillin/streptomycin | FUJIFILM Wako | Cat#168-23191 |
| Polyethylenimine | Polysciences | Cat#24765 |
| ReverTra Ace | Toyobo | Cat#TRT-101 |
| KOD ONE | Toyobo | Cat#KMM-101 |
| QuantumDye v3.1 | Digital Biology | Cat#QSD3 |
| Hi-Di Formamide | Thermo | Cat#4401457 |
| MES | Dojindo | Cat#345-01625 |
| HEPES | Dojindo | Cat#H0396 |
| Nigericin | AdipoGen | Cat#AG-CN2-0020 |
| BSA | FUJIFILM Wako | Cat#015-27053 |
| Taq Pro Universal SYBR qPCR Master Mix | Vazyme | Cat#Q712-02 |
| Tris | FUJIFILM Wako | Cat#207-06275 |
| SDS | FUJIFILM Wako | Cat#194-13985 |
| urea | FUJIFILM Wako | Cat#217-00615 |
| EDTA | DOJINDO | Cat#345-01865 |
| NaCl | FUJIFILM Wako | Cat#191-01665 |
| KCl | FUJIFILM Wako | Cat#163-03545 |
| Na-gluconate | FUJIFILM Wako | Cat#193-13195 |
| K-gluconate | FUJIFILM Wako | Cat#169-11835 |
| Mannitol | FUJIFILM Wako | Cat#133-00845 |
| Luminate Forte Western HRP Substrate | Merck Millipore | Cat#WBLUF0100 |
| Triton-X | Nakarai Tesque | Cat#28229-25 |
| Lipofectamine RNAi MAX | ThermoFisher | Cat#13778150 |
| PVDF membrane | Merck Millipore | Cat#IPVH00010 |
| Agmatine | Tokyo Chemical Industry | Cat#A0310 |
| Cadaverine | FUJIFILM Wako | Cat#350-28891 |
| Putrescine | Tokyo Chemical Industry | Cat#P13208 |
| Spermidine | Tokyo Chemical Industry | Cat#S0385 |
| Spermine | Tokyo Chemical Industry | Cat#S0069 |
| Adrenaline | Tokyo Chemical Industry | Cat#A0175 |
| Dopamine | Tokyo Chemical Industry | Cat#A0305 |
| Norepinephrine | Tokyo Chemical Industry | Cat#A0906 |
| Serotonine | Tokyo Chemical Industry | Cat#S0370 |
| Choline | FUJIFILM Wako | Cat#037-09815 |
| Histamine | Tokyo Chemical Industry | Cat#H0146 |
| Cimetidine | FUJIFILM Wako | Cat#036-16311 |
| Clonidine | FUJIFILM Wako | Cat#034-14293 |
| Decynium-22 | Tokyo Chemical Industry | Cat#D4486 |
| Desipramine | Sigma-Aldrich | Cat#D3900 |
| Diltiazem | FUJIFILM Wako | Cat#047-20311 |
| Diphenhydramine | FUJIFILM Wako | Cat#046-19771 |
| Estrone-3-sulfate | Sigma-Aldrich | Cat#E9505 |
| Guanidine | FUJIFILM Wako | Cat#074-05005 |
| Imipramine | FUJIFILM Wako | Cat#090-02601 |
| L-Arginine | Sigma-Aldrich | Cat#A5131 |
| L-carnitine | FUJIFILM Wako | Cat#327-64801 |
| L-ornitine | FUJIFILM Wako | Cat#156-00371 |
| Metformine | Sigma-Aldrich | Cat#270-432 |
| Methotrexate | FUJIFILM Wako | Cat#139-13571 |
| PAH | Cayman chemical | Cat#23726 |
| Procainamide | Sigma-Aldrich | Cat#P9391 |
| Quinidine | Sigma-Aldrich | Cat#01605KR |
| Quinine | Sigma-Aldrich | Cat#Q1125 |
| TEA | Sigma-Aldrich | Cat#T2265 |
| Verapamil | FUJIFILM Wako | Cat#222-00781 |
|  |  |  |
| Critical commercial assays |  |  |
| Protein Assay BCA Kit | FUJIFILM Wako | Cat#297-73101 |
|  |  |  |
| Cell lines |  |  |
| COS-7 | RIKEN BRC | Cat#RCB0539 |
| Caco-2 | RIKEN BRC | Cat#RCB0988 |
| THP-1 | Tohoku University* | Cat#TKG0267 |
| HepG2 | Tohoku University* | Cat#TKG0205 |
| A549 | Tohoku University* | Cat#TKG0184 |
| HCT-15 | Tohoku University* | Cat#TKG0504 |
| HaCaT | Provided by German Cancer Research Center |  |
| HeLa | Tohoku University* | Cat#TKG0331 |
| HEp-2 | Tohoku University* | Cat#TKG0403 |
| DLD-1 | Tohoku University* | Cat#TKG0379 |
| HT-1080 | JCRB** | Cat# JCRB9113 |
| MCF-7 | Tohoku University* | Cat#TKG0479 |
| ME-180 | Tohoku University* | Cat#TKG0437 |
| KB | Tohoku University* | Cat#TKG0401 |
| A431 | Tohoku University* | Cat#TKG0182 |
| HuT78 | Tohoku University* | Cat#TKG0375 |
| T24 | Tohoku University* | Cat#TKG0443 |
| BeWo | Tohoku University* | Cat#TKG0567 |
| PC-3 | Tohoku University* | Cat#TKG0600 |
| K562 | Tohoku University* | Cat#TKG0210 |
| HL-60 | Tohoku University* | Cat#TKG0345 |
| Jurkat | Tohoku University* | Cat#TKG0209 |
| Caki-1 | Provided by Chiba University |  |
| HPB-ALL | Tohoku University* | Cat#TKG0199 |
| MOLT-4 | Tohoku University* | Cat#TKG0229 |
| * Tohoku University; Cell Resource Center for Biomedical Research, Institute of Development, Aging  and Cancer, Tohoku University  ** JCRB; Japanese Collection of Research Bioresources Cell Bank | | |
|  |  |  |
| Oligonucleotides |  |  |
| Oligo(dT)20 | Sigma-Aldrich | N/A |
| Negative control Silencer-Selected siRNA | Thermo | Cat#4390844 |
| Primers for cDNA amplification are listed in  Table S2 | This paper | N/A |
| Primers for the generation of the cDNAs for  mutants are listed in Table S3 | This paper | N/A |
| Primers for qPCR are listed in Table S4 | This paper | N/A |
| siRNA sequences are listed in Table S5 | This paper | N/A |
|  |  |  |
| Recombinant DNA |  |  |
| pCI-neo | Promega | Cat#E1841 |
|  |  |  |
| Software and algorithms |  |  |
| WinNonlin | Certara | https://www.certara.com/software/phoenix-winnonlin/ |
| SigmaStat | Grafiti | https://grafiti.com/sigmastat/ |

Table S2

Primers for cDNA amplification of ATP13A family transporters

| Transporter | PCR | Orientation | Sequence (5’ to 3’) |
| --- | --- | --- | --- |
| ATP13A1 | 1st | Forward  Reverese | CGGCCTTGTCTTTTCCGGTCTCAGT  TCCCAGCTCAGTCTTCCAAGGGCGAGA |
|  | 2nd | Forward  Reverese | GGCGCTAGCGATGGCGGCAGCGGCGGCGG  CTTGCGGCCGCCATCTCAGGAAGGCACTT |
| ATP13A2 | 1st | Forward  Reverese | CAGGTGCCCCGCCCGCGTCA  CTGCCACCCCTACGCGGGAT |
|  | 2nd | Forward  Reverese | ACGGAATTCCGGCATGAGCGCAGACAGCA  GCGTCTAGACTACCTCAGGGGGCCGGCG |
| ATP13A3 | 1st | Forward  Reverese | AAAGCTGTGCAAATATCAGGA  TAAACTGAACTAGTGCCATTCTGACA |
|  | 2nd | Forward  Reverese | GAACTCGAGGGTATGGACAGGGAAGAAAG  TTATCTAGAGCTATGTTATGGTGATGATT |
| ATP13A4 | 1st | Forward  Reverese | CATCCTGGAAGCGTCTGCAC  ACCATGATTTGATAGGTAGCCCCAA |
|  | 2nd | Forward  Reverese | GTACTCGAGATGGGACACTTTGAGAAGGG  GTTTCTAGATTCAGAGTTGTTCTTCATTG |
| ATP13A5 | 1st | Forward  Reverese | AGAAGAGATCCTCGCCGGTT  ATGTACGACGACAATTCTGATTACAGC |
|  | 2nd | Forward  Reverese | AGAGTCGACGATGGAAGAGAACAGTAAGA  GCAGCGGCCGCATTACAGCCTGGCCCAGAAA |

The solid, broken, double, and wavy lines represent the restriction sites for EcoRI, XhoI, XbaI, and NotI, respectively.

Table S3

Primers for the generation of the cDNAs for ATP13A3 and ATP13A4 mutants

| Transporter | Mutation | Orientation | Sequence (5’ to 3’) |
| --- | --- | --- | --- |
| ATP13A3 | D498N | Forward  Reverese | TTTGCTTTAACAAGACTGGAACTCTAA  AGTCTTGTTAAAGCAAACAAGATTGAG |
| ATP13A4 | D486N | Forward  Reverese | TGCTTTAACAAGACAGGCACCTTAA  TCTTGTTAAAGCAGACAAGGTTTAA |

Table S4

Primers for FLAG-tag insertion into the extracellular loop of ATP13A3 and ATP13A4

| Transporter | Orientation | Sequence (5’ to 3’) |
| --- | --- | --- |
| ATP13A3 | Forward  Reverese | GACTACAAGGACGACGATGACAAGTTTTGGAATTCTTCACAC  CGTCGTCCTTGTAGTCCCCGCTTCCTGTTGTATT |
| ATP13A4 | Forward  Reverese | GACTACAAGGACGACGATGACAAGTCAGAGTTAACCATGTCT  CGTCGTCCTTGTAGTCGATGCTTTCATTTTGTAC |

Table S5

Primers for the real-time PCR analyses of the expression of ATP13A3 and ATP13A4

| Protein | Orientation | Sequence (5’ to 3’) |
| --- | --- | --- |
| ATP13A3 | Forward  Reverese | TGACATGGTGGCAACTCATAGT  CTGGCACAAGGTCGGTAGAAA |
| ATP13A4 | Forward  Reverese | GAGCCATAAGAAAGCCAGACC  TCTCTTGTCAAGCCTGATCCAA |
| UBC | Forward  Reverese | CTGGAAGATGGTCGTACCCTG  GGTCTTGCCAGTGAGTGTCT |
| GAPDH | Forward  Reverese | CGGAGTCAACGGATTTGGTCGTAT  AGCCTTCTCCATGGTGGTGAAGAC |

Table S6

Sequences of the siRNAs for ATP13A3 and ATP13A4

| Transporter | siRNA | Orientation | Sequence (5’ to 3’) |
| --- | --- | --- | --- |
| ATP13A3 | #1 | Sense  Antisense | GGCAACUCAUAGUACCGUATT  UACGGUACUAUGAGUUGCCAC |
|  | #2 | Sense  Antisense | CGUAGACAAUGAAACCGAATT  UUCGGUUUCAUUGUCUACGTG |
| ATP13A4 | #1 | Sense  Antisense | CACUAGUUUUGUUAGCGAATT  UUCGCUAACAAAACUAGUGGG |
|  | #2 | Sense  Antisense | CCACUAGUUUUGUUAGCGATT  UCGCUAACAAAACUAGUGGGT |
